# Reduced Models for Ferromagnetic Thin Films with Periodic Surface Roughness

**DOI:** 10.1007/s00332-017-9416-z

**Published:** 2017-10-11

**Authors:** M. Morini, V. Slastikov

**Affiliations:** 1 Università di Parma, Parma, Italy; 2 School of Mathematics, University of Bristol, Bristol, UK

**Keywords:** Micromagnetics, $\Gamma$-convergence, Homogenization, Dimension reduction, 35B27, 82D40, 49S05

## Abstract

We investigate the influence of periodic surface roughness in thin ferromagnetic films on shape anisotropy and magnetization behavior inside the ferromagnet. Starting from the full micromagnetic energy and using methods of homogenization and $\Gamma$-convergence, we derive a two-dimensional local reduced model. Investigation of this model provides an insight into the formation mechanism of *perpendicular magnetic anisotropy* and uniaxial anisotropy with an *arbitrary* preferred direction of magnetization.

## Introduction

Magnetic anisotropy is one of the fundamental properties of ferromagnetic materials. It is responsible for defining preferred directions of magnetization inside the ferromagnet. The main sources of magnetic anisotropy are magnetocrystalline anisotropy, prescribed by the crystalline structure of the material, and shape anisotropy, induced by the demagnetizing (or stray) field generated by the magnetization distribution inside the ferromagnet. In bulk ferromagnets, the magnetocrystalline anisotropy provides the leading contribution to magnetic anisotropy and the demagnetizing field is mainly responsible for formation of multiple domains inside the magnetic sample. On the other hand, in ferromagnetic nanostructures of reduced dimension (thin films, ribbons, nanowires, nanodots) stray field effects may dominate magnetocrystalline anisotropy and become the leading mechanism for choosing preferred magnetization direction.

The geometry of a ferromagnet plays a crucial role in defining the shape anisotropy. It has been observed that in flat ferromagnetic thin films the magnetization vector prefers to be constrained to the plane of the film and align tangentially to the boundary of the film (Aharoni 2001; Gay and Richter 1986; Gioia and James 1997; Kohn and Slastikov 2005). Recent micromagnetic studies of ferromagnetic thin layers, ribbons, and shells with nontrivial curvature of the surface of the film indicate that surface curvature has a significant effect on shape anisotropy, and in ferromagnetic thin structures with nonzero curvature magnetization prefers to be tangent to the surface (Carbou 2001; Gaididei et al. 2017, 2014; Sheka et al. 2015; Streubel et al. 2016). Therefore, the dominating effect of the shape anisotropy induced by the stray field is to *align magnetization direction tangentially to the surface* of the ferromagnetic nanostructure. This general principle works very well when surface variations happen on a scale larger than the thickness of the film (inverse surface curvature is larger than thickness). However, in the case of rapidly modulated surface, when inverse curvature is of the same order as the thickness of the film, the situation might be different and magnetic anisotropy, dominated by surface curvature effects, may produce preferred directions not tangential to the surface of the film (Bruno 1988; Chappert and Bruno 1988; Tretiakov et al. 2017). This behavior might be observed in ultrathin ferromagnetic films with the thickness reaching several monolayers, where the surface roughness can be comparable in amplitude and modulation to the thickness of the film, effectively leading to the large curvature of the film surface.

**Fig. 1:**
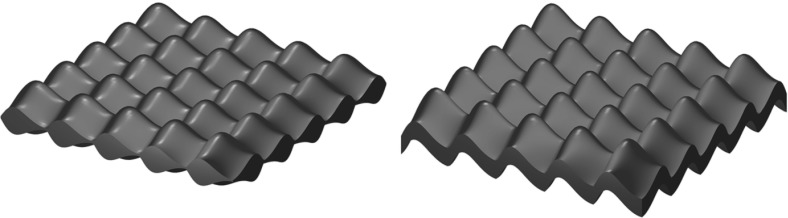
Thin film with generic periodic roughness $V_\varepsilon$ (left) and parallel roughness (right) (Tretiakov et al. 2017)

In this paper, we would like to understand the influence of the large surface curvature (or surface roughness) of thin films on the shape anisotropy induced by magnetostatic interaction. We consider the case of periodically modulated thin film surfaces modeling the surface roughness (see Fig. 1). In our study, we use the standard continuum model of micromagnetics (Aharoni 2001; Hubert and Schäfer 1998). In this framework, stable magnetization distributions inside a ferromagnet correspond to local minimizers of the micromagnetic energy which after a suitable nondimensionalization has the following form

1.1 $$\begin{aligned} {{\mathcal {E}}}(M) = d^2 \int _\Omega |\nabla M|^2 + K \int _\Omega \phi (M) + \int _{{{\mathbb {R}}}^3} |\nabla u|^2 - 2\int _{\Omega } h_\mathrm{ext} \cdot M. \end{aligned}$$

Here $\Omega \subset {\mathbb {R}}^3$ is the region occupied by a ferromagnet, $M :\Omega \rightarrow {\mathbb {S}}^2$ is the magnetization distribution, and the function *u* is defined on ${\mathbb {R}}^3$ and satisfies the following equation

1.2 $$\begin{aligned} \text{ div }\,(\nabla u + M \chi (\Omega )) = 0 \hbox { in } {\mathbb {R}}^3, \end{aligned}$$

with $\chi (\Omega )$ being the indicator of the set $\Omega$. The applied field is defined by $h_\mathrm{ext}$, and $\phi$ is the internal anisotropy function. Material parameters *d* and *K* correspond to an effective exchange and anisotropy constants, respectively. The four terms of the energy are known as exchange, anisotropy, magnetostatic and Zeeman energies, respectively. Due to the nonconvex and nonlocal nature this variational problem cannot be addressed in its full generality by current analytical methods.

The standard route to analytically investigate micromagnetic energy (1.1) is to consider a range of material and geometric parameters of a ferromagnet where the full three-dimensional model can be reduced to a simpler energy functional, capturing the essence of the magnetization behavior in ferromagnetic sample (DeSimone et al. 2006). The derivation and study of the reduced micromagnetic models is by no means a trivial task, but, in general, it is easier than investigation of the full three-dimensional model. Reduced models have been successfully derived and implemented to explore many magnetic phenomena in ferromagnetic nanostructures, including nanodots (Desimone 1995; Slastikov 2010), nanowires (Harutyunyan 2016; Kühn 2007; Sanchez 2009; Slastikov and Sonnenberg 2012), thin films (Carbou 2001; DeSimone et al. 2006, 2002; Gioia and James 1997; Kohn and Slastikov 2005), and curved structures of reduced dimensions (Carbou 2001; Gaididei et al. 2017, 2014; Sheka et al. 2015; Slastikov 2005).

The main goal of this paper is to obtain a comprehensive reduced model to describe the magnetization behavior in ferromagnetic thin films with periodic surface roughness. We concentrate on a regime where the thickness of the film is comparable to the amplitude and the period of thin film surface modulation and derive an effective *local* two-dimensional model. This reduced model has been examined, both analytically and numerically, in the recent paper (Tretiakov et al. 2017) and lead to some interesting observations. In particular, it was shown that in the special case of *parallel roughness*, when top and bottom surfaces of the layer are parallel, an extreme geometry is responsible for creating a strong uniaxial shape anisotropy with an *arbitrary* preferred direction depending on the surface roughness. This is a rather unexpected outcome suggesting that in certain regimes a surface roughness in ultrathin ferromagnetic films might lead to a *perpendicular magnetic anisotropy* (Chappert and Bruno 1988; Johnson et al. 1996; Vaz et al. 2008). In the case of more general roughness, when top and bottom surfaces are different, several examples have been also considered where instead the magnetization prefers to stay in-plane.

The dimension reduction problems for thin films with periodic surfaces or edges have been extensively studied in the mathematical community in the case where the energy functional has a local energy density, see, e.g., (Arrieta and Pereira 2011; Arrieta and Villanueva-Pesqueira 2017; Braides et al. 2000; Neukamm 2010; Neukamm and Velčić 2013). The existing results are not directly applicable in our setting due to the nonlocal nature of the stray field energy and one of the main difficulties in our case comes from homogenizing the magnetostatic contribution. In order to treat the magnetostatic energy, we first identify its leading contribution coming from dipolar interaction of charges at the top and bottom surfaces of thin film. This leading contribution can be represented as an integral with the kernel becoming singular in the limit of vanishing thickness (Kohn and Slastikov 2005). We investigate the homogenized limit of this singular integral and show that the leading order contribution has a local energy density [similar to the case of flat thin films, see (Gioia and James 1997)].

Using methods of $\Gamma$-convergence and two-scale convergence (Allaire 1992; Maso 1993), we obtain the limiting behavior of the full micromagnetic energy. Although the treatment of the exchange energy could be done using the framework of Braides et al. (2000), we cannot explicitly use their results due to the more general roughness considered in our paper. Therefore, we adopt the two-scale convergence approach adapted to dimension reduction problems as developed in Neukamm (2010) and provide a relatively simple self-contained proof of the $\Gamma$-convergence of the exchange energy. Special care has to be taken due to the fact that the magnetization distribution has values on a two-dimensional sphere.

The paper is organized as follows. In Sect. 2, we provide a rigorous mathematical formulation of the problem and state our main results in Theorem 2.2. Section 3 is devoted to the proof of Theorem 2.2. We begin our exposition in Sect. 3.1 by finding the limiting behavior of the magnetostatic energy in the case of “parallel roughness,” i.e., when the top and bottom surfaces of the film are exactly the same up to a shift in the vertical direction. The limiting behavior of the magnetostatic energy in the general case is treated in Sect. 3.2. After that, in Sect. 3.3 we identify the limiting behavior of the exchange energy. Combining all of the above, we arrive at the $\Gamma$-convergence result which completes the proof of Theorem 2.2 in Sect. 3.4.

## Formulation of the Problem and Statement of the Main Results

In this section, we provide a rigorous mathematical setup of the problem and state our main results in Theorem 2.2. We are interested in proving a $\Gamma$-convergence result and deriving a simplified reduced micromagnetic model [see (2.5)]. Without loss of generality, we are going to consider the case of zero anisotropy and external field, $K=0$ and $h_\mathrm{ext}=0$ since $\Gamma$-convergence is insensitive to continuous perturbations of the energy functional.

In the following, in order to indicate the generic point $x\in {\mathbb {R}}^3$ we will use the notation $x=(x', x_3)$, with $x'=(x_1, x_2)\in {\mathbb {R}}^2$ and $x_3\in {\mathbb {R}}$. We also set $Q:=(0,1) \times (0,1)$ and ${\mathbb {S}}^2=\partial B(0,1)=\{\xi \in {\mathbb {R}}^3:\, |\xi |=1\}$.

Let $f_1, f_2:{\mathbb {R}}^2\rightarrow (0, +\infty )$ be Lipschitz continuous *Q*-periodic functions, with periodic cell given by *Q*, with $f_1<f_2$, and $\omega \subset {\mathbb {R}}^2$ a bounded open set with Lipschitz boundary.

We will consider three-dimensional thin film domains with oscillating profiles of the form

2.1 $$\begin{aligned} V_\varepsilon =\left\{ (x',x_3) \, : \, x' \in \omega \, , \varepsilon f_1\left( \frac{x'}{\varepsilon }\right)<x_3< \varepsilon f_2\left( \frac{x'}{\varepsilon }\right) \right\} . \end{aligned}$$

We recall that given a *magnetization* $M\in H^1(V_\varepsilon ; {\mathbb {S}}^2)$, the corresponding micromagnetic energy of the film is defined as

2.2 $$\begin{aligned} {{\mathcal {E}}}_\varepsilon (M): = d^2 \int _{V_\varepsilon } |\nabla M|^2 + \int _{{\mathbb {R}}^3} |\nabla u|^2, \end{aligned}$$

where $d>0$ is a material parameter, the so-called *exchange constant*, and $u_\varepsilon$ is determined as the unique solution to

2.3 $$\begin{aligned} \Delta u = \mathrm{div} ( M \chi _{V_\varepsilon } ) \quad \text {in} {\mathbb {R}}^3 \end{aligned}$$

in ${\dot{H}}^1({\mathbb {R}}^3)$, that is, in the homogeneous Sobolev space obtained as a completion of $C^{\infty }_c({\mathbb {R}}^3)$ with respect to the norm $\Vert u\Vert _{{\dot{H}}^1({\mathbb {R}}^3)}:=\Vert \nabla u\Vert _{L^2({\mathbb {R}}^3)}$. In order to study the limiting behavior of the energy as $\varepsilon \rightarrow 0^+$, it is convenient to consider the following rescaled energies:

2.4 $$\begin{aligned} E_\varepsilon (m) = {d^2}\int _{\Omega _\varepsilon } \left( | \nabla _{x'} m|^2 + \frac{1}{\varepsilon ^2} |\partial _{x_3} m|^2 \right) \, {\hbox {d}}x+ \frac{1}{\varepsilon } \int _{{\mathbb {R}}^3} |\nabla u|^2, \end{aligned}$$

defined for all $m\in H^1(\Omega _\varepsilon ; {\mathbb {S}}^2)$, where

$$\begin{aligned} \Omega _\varepsilon = \left\{ (x', x_3) \, : \, x' \in \omega \, , f_1\left( \frac{x'}{\varepsilon }\right)<x_3< f_2\left( \frac{x'}{\varepsilon }\right) \right\} \end{aligned}$$

and *u* now solves (2.3) with $M\in H^1(V_\varepsilon ; {\mathbb {S}}^2)$ defined by

$$\begin{aligned} M(x',x_3):=m(x',x_3/\varepsilon )\,. \end{aligned}$$

Note that

$$\begin{aligned} E_\varepsilon (m)=\frac{1}{\varepsilon }{{\mathcal {E}}}_\varepsilon (M)\,. \end{aligned}$$

We also set

$$\begin{aligned} Q_{f_1,f_2}:=\{(x', x_3)\in {\mathbb {R}}^3: x'\in Q\text { and }f_1(x')<x_3<f_2(x')\} \end{aligned}$$

and denote by $H^1_\#(Q_{f_1,f_2}; {\mathbb {R}}^3)$ the space of functions $\varphi \in H^1(Q_{f_1,f_2}; {\mathbb {R}}^3)$ that are *Q*-periodic in the $x'$-variable. We will show that the limiting energy is given by the following functional $E_0: H^1(\omega ; {\mathbb {S}}^2)\rightarrow [0, +\infty )$ defined by

2.5 $$\begin{aligned} E_0(m):=d^2\int _{\omega }g_{\mathrm {hom}}(\nabla m)\, {\hbox {d}}x'+ \int _{\omega }A_{\mathrm {hom}}\, m \cdot m\, {\hbox {d}}x' \end{aligned}$$

for every $m\in H^1(\omega ; {\mathbb {S}}^2)$, where $g_{\mathrm {hom}}:{\mathbb {M}}^{3\times 2} \rightarrow {\mathbb {R}}$ is given by

2.6 $$\begin{aligned} g_{\mathrm {hom}}(\xi ):=\inf _{\varphi \in H^1_{\#}(Q_{f_1,f_2};\,{\mathbb {R}}^3)}\int _{Q_{f_1,f_2}}\left( |\xi +\nabla _{y'}\varphi |^2+|\partial _{y_3}\varphi |^2\right) \, {\hbox {d}}y \end{aligned}$$

and *constant matrix* $A_{\mathrm {hom}}$ is defined as

2.7 $$\begin{aligned}&A_{\mathrm {hom}}:= \quad \nonumber \\&\frac{1}{4\pi }\int _{Q}\int _{{\mathbb {R}}^2} \Biggl ( \frac{n_1(x') \otimes n_1(z'+x')}{\sqrt{|z'|^2 + \left| f_1( z'+x') - f_1(x') \right| ^2}} - \frac{n_1(x') \otimes n_1(z'+x')}{\sqrt{|z'|^2 +1}} \Biggr )\, {\hbox {d}}z'{\hbox {d}}x' \nonumber \\&\quad +\frac{1}{4\pi }\int _{Q}\int _{{\mathbb {R}}^2}\Biggl ( \frac{n_2(x') \otimes n_2(z'+x') }{\sqrt{|z'|^2 + \left| f_2( z'+x') - f_2(x') \right| ^2}} - \frac{n_2(x') \otimes n_2(z'+x') }{\sqrt{|z'|^2 +1}} \Biggr )\, {\hbox {d}}z'{\hbox {d}}x' \nonumber \\&\quad - \frac{1}{2\pi }\int _{Q}\int _{{\mathbb {R}}^2} \Biggl ( \frac{n_1(x') \otimes n_2(z'+x')}{\sqrt{|z'|^2 + \left| f_2( z'+x') - f_1(x') \right| ^2}} - \frac{n_1(x') \otimes n_2(z'+x')}{\sqrt{|z'|^2 +1}} \Biggr )\, {\hbox {d}}z'{\hbox {d}}x' \nonumber \\&\quad +\frac{1}{4\pi }\int _{Q}\int _{{\mathbb {R}}^2}\Biggl (\frac{I-\mathbf {e_3}\otimes \mathbf {e_3}}{(|z'|^2+1)^{3/2}} -\frac{3(z',0)\otimes (z',0)}{(|z'|^2+1)^{5/2}}\Biggr ) \nonumber \\&\quad \cdot (f_2(z'+x')-f_1(z'+x'))(f_2(x')-f_1(x'))\, {\hbox {d}}z'{\hbox {d}}x'. \end{aligned}$$

In the above formula, we used the notation

2.8 $$\begin{aligned} n_i(x'):=(-\nabla f_i(x'), 1)\quad i=1,2\qquad \text {and}\qquad \mathbf {e_3}:=(0,0,1)\,. \end{aligned}$$

We will also show below (see Sect. 3.1) that in the case of parallel profiles, that is when $f_2= f_1+a$ for a suitable constant $a>0$ (see Fig. 1) the expression of $A_{\mathrm {hom}}$ reduces to the following much simpler formula:

2.9 $$\begin{aligned} A_{\mathrm {hom}}=\frac{1}{2\pi } \int _Q \int _{{\mathbb {R}}^2}&\Biggl [ \frac{ n(x') \otimes n(z'+x')}{\sqrt{|z'|^2 + \left| f( z'+x') - f(x') \right| ^2}}\nonumber \\&\quad - \frac{ n(x') \otimes n(z'+x')}{\sqrt{|z'|^2 + \left| a+ f(z'+x') - f( x') \right| ^2}} \Biggr ]\, {\hbox {d}} z' {\hbox {d}} x', \end{aligned}$$

with

$$\begin{aligned} n(x'):=(-\nabla f(x'), 1)\,. \end{aligned}$$

### Remark 2.1

We note that the geometry of the profiles, that is the shape of $f_1$ and $f_2$, influences the properties of $g_{\mathrm {hom}}$ and $A_{\mathrm {hom}}$ defined in (2.6) and (2.7). The general problem of analytically investigating the properties of $A_{\mathrm {hom}}$ and $g_{\mathrm {hom}}$ turns out to be quite challenging. For some heuristic and numerical observations, we refer to Tretiakov et al. (2017).

The link between (2.4) and (2.5) is made precise by the following compactness and $\Gamma$-convergence type statement, which represents the main result of the paper.

### Theorem 2.2

The following statements hold.

(i) (Compactness) Let $\{m_\varepsilon \}_\varepsilon$ be such that $m_\varepsilon \in H^1(\Omega _\varepsilon ; {\mathbb {S}}^2)$ for every $\varepsilon >0$ and
  $$\begin{aligned} \sup _{\varepsilon } E_\varepsilon (m_\varepsilon )<+\infty . \end{aligned}$$
  Then, there exists $m_0\in H^1(\omega ; {\mathbb {S}}^2)$ and a (not relabeled) subsequence such that
  2.10 $$\begin{aligned} \int _{\Omega _\varepsilon }|m_\varepsilon (x)-m_0(x')|^2\, \mathrm{d}x\rightarrow 0 \end{aligned}$$
  as $$\varepsilon \rightarrow 0^+$$.
(ii) ($\Gamma$-liminf inequality) Let $m_0\in H^1(\omega ; {\mathbb {S}}^2)$ and let $\{m_\varepsilon \}_\varepsilon$ be such that $m_\varepsilon \in H^1(\Omega _\varepsilon ; {\mathbb {S}}^2)$ for every $\varepsilon >0$ and (2.10) holds. Then
  $$\begin{aligned} E_0(m_0)\le \liminf _{\varepsilon \rightarrow 0} E_\varepsilon (m_\varepsilon )\,. \end{aligned}$$
(iii) ($\Gamma$-limsup inequality) For any $m_0\in H^1(\omega ; {\mathbb {S}}^2)$, there exists $\{m_\varepsilon \}_\varepsilon$, with $m_\varepsilon \in H^1(\Omega _\varepsilon ; {\mathbb {S}}^2)$ for all $\varepsilon >0$, such that (2.10) holds and
  $$\begin{aligned} E_0(m_0)= \lim _{\varepsilon \rightarrow 0} E_\varepsilon (m_\varepsilon )\,. \end{aligned}$$

As a consequence of the above theorem, we will be able to establish the following corollary about the asymptotic behavior of global minimizers.

### Corollary 2.3

Let $m_\varepsilon \in H^1(\Omega _\varepsilon ; {\mathbb {S}}^2)$ be a minimizer of $E_\varepsilon$. Then, up to a (not relabeled) subsequence,

$$\begin{aligned} \int _{\Omega _\varepsilon }|m_\varepsilon -e_0|^2\, {\hbox {d}}x\rightarrow 0 \end{aligned}$$

for a suitable $e_0\in {\mathbb {S}}^2$ such that

$$\begin{aligned} A_{\mathrm {hom}}\, e_0\cdot e_0=\min _{e\in {\mathbb {S}}^2} A_{\mathrm {hom}}\, e\cdot e\,. \end{aligned}$$

## Proofs of the Results

In this section, we collect the proofs of the main results. We treat separately the magnetostatic and the exchange energies. We start with the study of the magnetostatic energy, which represents the main novelty of the present analysis. In order to simplify the exposition, in Sect. 3.1 we consider first the case of parallel profiles (see Fig. 1). Then, in Sect. 3.2 we consider the case of general surface roughness, requiring a more intricate analysis, and identify the limiting behavior of the magnetostatic energy in Proposition 3.13. The $\Gamma$-limit of the exchange energy is investigated in Sect. 3.3 (see Propositions 3.16, 3.21). Finally, combining the aforementioned results we provide the proof of Theorem 2.2 in Sect. 3.4.

### Study of the Magnetostatic Energy: The Case of Parallel Profiles

Following Kohn and Slastikov (2005), Slastikov (2005), in order to treat the magnetostatic energy we show that its limiting behavior can be reduced to that of the energy of magnetic charges at the top and bottom surfaces of the thin layer (see Lemmas 3.1–3.5). We utilize some results proven in Kohn and Slastikov (2005), see Lemma 3.1 and Lemma 3.2; however, due to the presence of the two scales, it is necessary to provide self-contained proofs for Lemma 3.4 and Lemma 3.5. The core of the analysis is then represented by the study of the leading order contribution of the magnetostatic energy (see Proposition 3.9). The main new difficulties are related to the fact that there is a nontrivial interaction between the homogenization and the dimension reduction processes in the limiting singular behavior of the integral kernel coming from the magnetostatic energy.

In what follows we set $f_1=f$ and $f_2=f+a$, for some *Q*-periodic Lipschitz continuous function *f* and $a>0$, so that (2.1) becomes

3.1 $$\begin{aligned} V_\varepsilon =\left\{ (x',x_3) \, : \, x' \in \omega \, , \varepsilon f\left( \frac{x'}{\varepsilon }\right)<x_3< a \varepsilon +\varepsilon f\left( \frac{x'}{\varepsilon }\right) \right\} \end{aligned}$$

and thus

$$\begin{aligned} \Omega _\varepsilon = \left\{ (x', x_3) \, : \, x' \in \omega \, , f\left( \frac{x'}{\varepsilon }\right)<x_3< a + f\left( \frac{x'}{\varepsilon }\right) \right\} \,. \end{aligned}$$

The typical examples that we might consider is

$$\begin{aligned} f(x')= \sin ^2(\pi x_1)\sin ^2(\pi x_2) \quad \hbox { or } \quad f(x')= \sin ^2(\pi x_1)\,. \end{aligned}$$

We start by recalling the following well-known useful representation formula for the *magnetostatic* energy.

#### Lemma 3.1

Let *u* solve (2.3). Then

3.2 $$\begin{aligned} 4\pi \int _{{\mathbb {R}}^3}|\nabla u|^2\, {\hbox {d}}x&= \int _{V_\varepsilon }\int _{V_\varepsilon }\frac{1}{|x-y|}\text{ div }\,M(x)\text{ div }\,M(y)\, \mathrm{d}x\mathrm{d}y\nonumber \\&\quad +\int _{\partial V_\varepsilon }\int _{\partial V_\varepsilon } \frac{1}{|x-y|}(M\cdot \nu _{\varepsilon })(x)(M\cdot \nu _{\varepsilon })(y)\, \mathrm{d}{{\mathcal {H}}}^{2}(x)\mathrm{d}{{\mathcal {H}}}^2(y)\\&\quad -2\int _{\partial V_\varepsilon }\int _{V_\varepsilon }\frac{1}{|x-y|}\text{ div }\,M(y)(M\cdot \nu _\varepsilon )(x)\, \mathrm{d}y \mathrm{d}{{\mathcal {H}}}^{2}(x)\,, \nonumber \end{aligned}$$

where $\nu _\varepsilon$ denotes the outer unit normal to $V_\varepsilon$.

#### Proof

See [Kohn and Slastikov (2005), p 237]. $\square$

*Notational warning* In all the following results (and proofs) *C* will denote a positive constant possibly depending only on *f* and $\omega$ (and possibly changing from line to line).

The next lemma provides a simple estimate that will allow us to reduce to the case of $x_3$-independent magnetizations.

#### Lemma 3.2

Let $$M\in H^1(V_\varepsilon ; {\mathbb {S}}^2)$$. Set

$$\begin{aligned} {\overline{M}}(x'):=\frac{1}{a \varepsilon }\int _{\varepsilon f(x'/\varepsilon )}^{\varepsilon a+ \varepsilon f(x'/\varepsilon )}M(x', x_3)\, \mathrm{d}x_3 \end{aligned}$$

and let ${\bar{u}}$ be the solution to (2.3) with *M* replaced by ${\overline{M}}$. Then,

$$\begin{aligned} \biggl | \int _{{\mathbb {R}}^3}|\nabla u|^2\, \mathrm{d}x -\int _{{\mathbb {R}}^3}|\nabla {\bar{u}}|^2\, \mathrm{d}x\biggr |\le C \varepsilon ^{3/2}\biggl \Vert \frac{\partial M}{\partial x_3}\biggr \Vert _{L^2(V_\varepsilon )}\,. \end{aligned}$$

#### Proof

The proof can be established arguing as in Kohn and Slastikov (2005), Lemma 3. $\square$

#### Remark 3.3

The previous lemma holds also in the general case (2.1) with the same proof.

In the next two lemmas, we estimate the first and the third terms, respectively, of the representation formula (3.2). We show that these terms vanish in the limit as $\varepsilon \rightarrow 0$ and do not contribute to the reduced energy.

#### Lemma 3.4

Under the hypothesis and with the notation of the previous lemma, we have

$$\begin{aligned} \biggl |\int _{V_\varepsilon }\int _{V_\varepsilon }\frac{1}{|x-y|}\text{ div }\,{\overline{M}}(x')\text{ div }\,{\overline{M}}(y')\, \mathrm{d}x\mathrm{d}y\biggr |\le C\varepsilon ^2\Vert \text{ div }\,_{x'}{\overline{M}}\Vert ^2_{L^2(\omega )}\,. \end{aligned}$$

#### Proof

Using the fact that ${\overline{M}}$ is independent of $x_3$, one immediately gets

$$\begin{aligned}&\biggl |\int _{V_\varepsilon }\int _{V_\varepsilon }\frac{1}{|x-y|}\text{ div }\,{\overline{M}}(x')\text{ div }\,{\overline{M}}(y')\, \mathrm{d}x\mathrm{d}y\biggr |\\&\quad \le a^2\varepsilon ^2 \int _{\omega }\int _{\omega }\frac{1}{|x'-y'|}|\text{ div }\,_{x'} {\overline{M}}(x')|\, |\text{ div }\,_{y'} {\overline{M}}(y')|\, \mathrm{d}x'\mathrm{d}y' \le C\varepsilon ^2\Vert \text{ div }\,_{x'}{\overline{M}}\Vert ^2_{L^2(\omega )}\,, \end{aligned}$$

where the last estimate follows from the generalized Young’s inequality (see Lieb and Loss 2010). $\square$

#### Lemma 3.5

With the notation of the previous lemma, we have

$$\begin{aligned} \biggl |\int _{\partial V_\varepsilon }\int _{V_\varepsilon }\frac{1}{|x-y|}\text{ div }\,{\overline{M}}(y')({\overline{M}}\cdot \nu _\varepsilon )(x')\, \mathrm{d}y \mathrm{d}{{\mathcal {H}}}^{2}(x)\biggr | \le C\varepsilon ^{3/2}\Vert \text{ div }\,_{x'} {\overline{M}} \Vert _{L^2(\omega )}\,. \end{aligned}$$

#### Proof

Using the inequality

3.3 $$\begin{aligned} \frac{1}{\sqrt{|x'-y'|^2+(x_3-y_3)^2}}\le \frac{1}{\sqrt{|x'-y'|}\sqrt{|x_3-y_3|}} \end{aligned}$$

and setting

$$\begin{aligned}&A:=\biggl |\int _{\omega }\int _{\varepsilon f(y'/\varepsilon )}^{a\varepsilon + \varepsilon f(y'/\varepsilon )}\int _{\omega }\frac{\text{ div }\,{\overline{M}}(y')({\overline{M}}(x')\cdot (-\nabla f(x'/\varepsilon ), 1))}{\sqrt{(x'-y')^2+(\varepsilon a+ \varepsilon f(x'/\varepsilon )-y_3)^2}}\mathrm{d}x'\mathrm{d}y_3\mathrm{d}y' \\&\qquad +\int _{\omega }\int _{\varepsilon f(y'/\varepsilon )}^{a\varepsilon + \varepsilon f(y'/\varepsilon )}\int _{\omega }\frac{\text{ div }\,{\overline{M}}(y')({\overline{M}}(x')\cdot (\nabla f(x'/\varepsilon ), -1))}{\sqrt{(x'-y')^2+( \varepsilon f(x'/\varepsilon )-y_3)^2}}\mathrm{d}x'\mathrm{d}y_3\mathrm{d}y'\biggr |\,, \end{aligned}$$

we have

3.4 $$\begin{aligned}&\biggl |\int _{\partial V_\varepsilon }\int _{V_\varepsilon }\frac{\text{ div }\,{\overline{M}}(y')({\overline{M}}\cdot \nu _\varepsilon )(x')}{|x-y|}\, \mathrm{d}y \mathrm{d}{{\mathcal {H}}}^{2}(x)\biggr | \nonumber \\&\quad \le \biggl |\int _{\omega }\int _{\partial \omega }\int _{\varepsilon f(x'/\varepsilon )}^{\varepsilon a+ \varepsilon f(x'/\varepsilon )}\int _{\varepsilon f(y'/\varepsilon )}^{\varepsilon a+ \varepsilon f(y'/\varepsilon )}\int \frac{\text{ div }\,{\overline{M}}(y')({\overline{M}}\cdot \nu _\varepsilon )(x')}{\sqrt{|x'-y'|^2+|x_3-y_3|^2}}\, \mathrm{d}y_3 \mathrm{d} x_3 \mathrm{d}{{\mathcal {H}}}^{1}(x')\mathrm{d}y'\biggr |+A\nonumber \\&\quad \le \int _{\omega }\int _{\partial \omega }\frac{|\text{ div }\,{\overline{M}}(y')|}{\sqrt{|x'-y'|}}\mathrm{d}{{\mathcal {H}}}^{1}(x')\mathrm{d}y' \int _{0}^{\varepsilon a+ \Vert f\Vert _\infty \varepsilon }\int _{0}^{\varepsilon a+ \Vert f\Vert _\infty \varepsilon }\frac{1}{\sqrt{|x_3-y_3|}}\, \mathrm{d}y_3 \mathrm{d} x_3 +A\nonumber \\&\quad \le C \varepsilon ^{3/2} \int _{\omega }\int _{\partial \omega }\frac{|\text{ div }\,{\overline{M}}(y')|}{\sqrt{|x'-y'|}}\mathrm{d}{{\mathcal {H}}}^{1}(x')\mathrm{d}y' +A \le C \varepsilon ^{3/2} \Vert \text{ div }\,_{x'} {\overline{M}} \Vert _{L^2(\omega )}+A\,. \end{aligned}$$

Since for $y_3\in (\varepsilon f(y'/\varepsilon ), a\varepsilon + \varepsilon f(y'/\varepsilon ))$ we may find $L>0$ large enough (depending only on *f* and *a*) so that

3.5 $$\begin{aligned}&\biggl | \frac{1}{\sqrt{(x'-y')^2+( \varepsilon f(x'/\varepsilon )-y_3)^2}} - \frac{1}{\sqrt{(x'-y')^2+(a \varepsilon + \varepsilon f(x'/\varepsilon )-y_3)^2}}\biggr | \nonumber \\&\quad \le \frac{1}{|x'-y'|} - \frac{1}{\sqrt{(x'-y')^2+ \varepsilon ^2 L^2}} =:K_\varepsilon (x'-y')\,, \end{aligned}$$

and we can estimate

$$\begin{aligned} A \le C\varepsilon \int _\omega \int _\omega |\text{ div }\,{\overline{M}}(y')| K_\varepsilon (x'-y')\,\mathrm{d}x' \mathrm{d}y'\,. \end{aligned}$$

In turn, by the generalized Young’s inequality and using the fact that

3.6 $$\begin{aligned} \int _{{\mathbb {R}}^2}K_\varepsilon (z')\, \mathrm{d}z'&=2\pi \int _0^{+\infty }r\left( \frac{1}{r}-\frac{1}{\sqrt{r^2+\varepsilon ^2L^2}}\right) \, \mathrm{d}r \nonumber \\&=2\pi \int _0^{+\infty }\frac{\varepsilon ^2 L^2}{\sqrt{r^2+\varepsilon ^2L^2}(\sqrt{r^2+\varepsilon ^2L^2}+r)}\, \mathrm{d}r\nonumber \\&\le 2\pi \int _0^\infty \frac{\varepsilon ^2 L^2}{r^2 + L^2 \varepsilon ^2} \mathrm{d}r =\pi ^2\varepsilon L \,, \end{aligned}$$

we obtain

$$\begin{aligned} A \le C\varepsilon \Vert K_\varepsilon \Vert _{L^1({\mathbb {R}}^2)} \Vert \text{ div }\,{\overline{M}} \Vert _{L^2(\omega )}\le C\varepsilon ^2 \Vert \text{ div }\,{\overline{M}} \Vert _{L^2(\omega )}\,. \end{aligned}$$

Combining the last inequality with (3.4), we conclude the proof of the lemma. $\square$

The estimates provided by the next two lemmas will be useful in the computing the limit of the second term in (3.2).

#### Lemma 3.6

With the same notation of the previous lemma, we have

$$\begin{aligned} \int _{\omega }\int _{\partial \omega }\int _{\varepsilon f(x'/\varepsilon )}^{a\varepsilon + \varepsilon f(x'/\varepsilon )}&\biggl |\frac{1}{\sqrt{|x'-y'|^2+(\varepsilon a+ \varepsilon f(x'/\varepsilon )-y_3)^2}}&\\&-\frac{1}{\sqrt{|x'-y'|^2+(\varepsilon f(x'/\varepsilon )-y_3)^2}}\biggl |\mathrm{d}y_3\mathrm{d}x'\mathrm{d}y'\le C\varepsilon ^2 \end{aligned}$$

#### Proof

We can estimate the integrand as in (3.5) and (3.6) to easily conclude. $\square$

#### Lemma 3.7

We have

$$\begin{aligned} \int _{\partial \omega }\int _{\partial \omega }\int _{\varepsilon f(x'/\varepsilon )}^{a\varepsilon + \varepsilon f(x'/\varepsilon )}\int _{\varepsilon f(y'/\varepsilon )}^{a\varepsilon + \varepsilon f(y'/\varepsilon )} \frac{1}{\sqrt{|x'-y'|^2+(x_3-y_3)^2}}\mathrm{d}y_3\mathrm{d}x_3\mathrm{d}{{\mathcal {H}}}^1(y')\mathrm{d}{{\mathcal {H}}}^1(x')\le C \varepsilon ^{3/2}\,. \end{aligned}$$

#### Proof

The proof is straightforward after recalling (3.3). $\square$

We will also need the following simple and rather standard result on the approximation of the identity. It is a particular case of a more general statement; however, we formulate it only in the form that serves our purposes.

#### Lemma 3.8

Let $( K_\varepsilon )$ be a family of nonnegative kernels satisfying

3.7 $$\begin{aligned}&\sup _{\varepsilon>0}\int _{{\mathbb {R}}^2} K_\varepsilon (z')\, \mathrm{d}z'=:M <+\infty \ \hbox { and for any fixed } \delta>0 \ \nonumber \\&\quad \int _{|{ z'}| > \delta } K_\varepsilon ({z'})\mathrm{d}z' \rightarrow 0 \hbox { as }\varepsilon \rightarrow 0\,. \end{aligned}$$

Let $u_\varepsilon \rightarrow u$ in $L^1({\mathbb {R}}^2; {\mathbb {R}}^3)$. Then

$$\begin{aligned} \int _{{\mathbb {R}}^2}\int _{{\mathbb {R}}^2}K_\varepsilon (x'-y')|u_\varepsilon (x')-u(y')|\, \mathrm{d}x'\mathrm{d}y'\rightarrow 0 \end{aligned}$$

as $$\varepsilon \rightarrow 0^+$$.

#### Proof

The proof is rather standard. Observe first that by (3.7) it easily follows that

3.8 $$\begin{aligned}&w\in C_c({\mathbb {R}}^2; {\mathbb {R}}^3)\Rightarrow \int _{{\mathbb {R}}^2}\int _{{\mathbb {R}}^2}K_\varepsilon (x'-y')|w(x')-w(y')|\, \mathrm{d}x'\mathrm{d}y'\rightarrow 0\nonumber \\&\quad \text { as }\varepsilon \rightarrow 0^+\,. \end{aligned}$$

Fix $\delta >0$ and find $w\in C_c({\mathbb {R}}^2; {\mathbb {R}}^3)$ and ${{\bar{\varepsilon }}}>0$ such that $\Vert w-u\Vert _{1}\le \delta$ and $\Vert u_\varepsilon -u\Vert _{1}\le \delta$ for all $\varepsilon \in (0, {{\bar{\varepsilon }}})$. Then for all such $\varepsilon$ we have

$$\begin{aligned} \int _{{\mathbb {R}}^2}&\int _{{\mathbb {R}}^2}K_\varepsilon (x'-y')|u_\varepsilon (x')-u(y')|\, \mathrm{d}x'\mathrm{d}y' \\&\le \int _{{\mathbb {R}}^2}\int _{{\mathbb {R}}^2}K_\varepsilon (x'-y')|u_\varepsilon (x')-u(x')|\, \mathrm{d}x'\mathrm{d}y'\\&\quad +2\int _{{\mathbb {R}}^2}\int _{{\mathbb {R}}^2}K_\varepsilon (x'-y')|u(x')-w(x')|\, \mathrm{d}x'\mathrm{d}y'\\&\quad +\int _{{\mathbb {R}}^2}\int _{{\mathbb {R}}^2}K_\varepsilon (x'-y')|w(x')-w(y')|\, \mathrm{d}x'\mathrm{d}y'\\&= \Vert K_\varepsilon \Vert _{1}\left( \Vert u_\varepsilon -u\Vert _{1}\right. \\&\quad \left. + 2\Vert w-u\Vert _{1}\right) +\int _{{\mathbb {R}}^2}\int _{{\mathbb {R}}^2}K_\varepsilon (x'-y')|w(x')-w(y')|\, \mathrm{d}x'\mathrm{d}y'\\&\le 3 M\delta +\int _{{\mathbb {R}}^2}\int _{{\mathbb {R}}^2}K_\varepsilon (x'-y')|w(x')-w(y')|\, \mathrm{d}x'\mathrm{d}y'\,, \end{aligned}$$

where in the last inequality we used the first assumption in (3.7). Recalling (3.8) we deduce

$$\begin{aligned} \limsup _{\varepsilon \rightarrow 0}\int _{{\mathbb {R}}^2}\int _{{\mathbb {R}}^2}K_\varepsilon (x'-y')|u_\varepsilon (x')-u(y')|\, \mathrm{d}x'\mathrm{d}y'\le 3M\delta \end{aligned}$$

and the conclusion follows by the arbitrariness of $\delta$. $\square$

The following proposition identifies the limit as $\varepsilon \rightarrow 0$ of the second term in (3.2), accounting for the interaction between the boundary charges, and represents the main brick in the proof of Theorem 2.2.

#### Proposition 3.9

Let $m_0\in L^2(\omega ; {\mathbb {S}}^2)$ and let $({\overline{M}}_\varepsilon ) \subset L^2(\omega ; {\mathbb {R}}^3)$ be such that $|{\overline{M}}_\varepsilon |\le 1$ for all $\varepsilon$ and ${\overline{M}}_\varepsilon \rightarrow m_0$ in $L^2(\omega ; {\mathbb {R}}^3)$. Then

$$\begin{aligned}&\frac{1}{4\pi \varepsilon }\int _{\partial V_\varepsilon }\int _{\partial V_\varepsilon } \frac{1}{|x-y|}({\overline{M}}_\varepsilon (x')\cdot \nu _{\varepsilon }(x))({\overline{M}}_\varepsilon (y')\cdot \nu _{\varepsilon }(y))\, \mathrm{d}{{\mathcal {H}}}^{2}(x)\mathrm{d}{{\mathcal {H}}}^2(y) \nonumber \\&\quad \rightarrow \int _\omega A_{\mathrm {hom}}\, m_0\cdot m_0\, \mathrm{d}x'\,, \end{aligned}$$

where $A_{\mathrm {hom}}$ is the constant matrix defined in (2.9).

#### Proof

We start by decomposing $\partial V_\varepsilon$ as $\partial V_\varepsilon =\Gamma ^+_\varepsilon \cup \Gamma ^-_\varepsilon \cup \Gamma ^\mathrm{lat}_\varepsilon$, with $\Gamma ^+_\varepsilon$ and $\Gamma ^-_\varepsilon$ denoting the top and the bottom part of $\partial V_\varepsilon$, respectively, and $\Gamma ^\mathrm{lat}_\varepsilon$ being the lateral boundary. Observe now that we may split the double integral $\int _{\partial V_\varepsilon }\int _{\partial V_\varepsilon }$ as

3.9 $$\begin{aligned} \int _{\partial V_\varepsilon }\int _{\partial V_\varepsilon }&= \int _{\Gamma ^+_\varepsilon }\int _{\Gamma ^+_\varepsilon }+\int _{\Gamma ^-_\varepsilon }\int _{\Gamma ^-_\varepsilon }+2\int _{\Gamma ^+_\varepsilon }\int _{\Gamma ^-_\varepsilon }+2\int _{\Gamma ^\mathrm{lat}_\varepsilon }\int _{\Gamma ^+_\varepsilon \cup \Gamma ^-_\varepsilon }+\int _{\Gamma ^\mathrm{lat}_\varepsilon }\int _{\Gamma ^\mathrm{lat}_\varepsilon }\nonumber \\&=2 \int _{\Gamma ^+_\varepsilon }\int _{\Gamma ^+_\varepsilon }+2\int _{\Gamma ^+_\varepsilon }\int _{\Gamma ^-_\varepsilon }+2\int _{\Gamma ^\mathrm{lat}_\varepsilon }\int _{\Gamma ^+_\varepsilon \cup \Gamma ^-_\varepsilon }+\int _{\Gamma ^\mathrm{lat}_\varepsilon }\int _{\Gamma ^\mathrm{lat}_\varepsilon }\,, \end{aligned}$$

where we used the obvious identity $\int _{\Gamma ^+_\varepsilon }\int _{\Gamma ^+_\varepsilon }=\int _{\Gamma ^-_\varepsilon }\int _{\Gamma ^-_\varepsilon }$, which follows from the fact that $\Gamma ^+_\varepsilon$ and $\Gamma ^-_\varepsilon$ are parallel. By Lemma 3.6, we easily get

3.10 $$\begin{aligned} \int _{\Gamma ^\mathrm{lat}_\varepsilon }\int _{\Gamma ^+_\varepsilon \cup \Gamma ^-_\varepsilon } \frac{1}{|x-y|}({\overline{M}}_\varepsilon (x')\cdot \nu _{\varepsilon }(x))({\overline{M}}_\varepsilon (y')\cdot \nu _{\varepsilon }(y))\, \mathrm{d}{{\mathcal {H}}}^{2}(x)\mathrm{d}{{\mathcal {H}}}^2(y)\le C \varepsilon ^2\,,\nonumber \\ \end{aligned}$$

while Lemma 3.7 yields

3.11 $$\begin{aligned} \int _{\Gamma ^{lat}_\varepsilon }\int _{\Gamma ^{lat}_\varepsilon } \frac{1}{|x-y|}({\overline{M}}_\varepsilon (x')\cdot \nu _{\varepsilon }(x))({\overline{M}}_\varepsilon (y')\cdot \nu _{\varepsilon }(y))\, \mathrm{d}{{\mathcal {H}}}^{2}(x)\mathrm{d}{{\mathcal {H}}}^2(y)\le C \varepsilon ^{3/2}.\nonumber \\ \end{aligned}$$

Thus, combining (3.9)–(3.11) we get

3.12 $$\begin{aligned} \lim _{\varepsilon \rightarrow 0}\frac{1}{4\pi \varepsilon }\int _{\partial V_\varepsilon }&\int _{\partial V_\varepsilon } \frac{1}{|x-y|}({\overline{M}}_\varepsilon (x')\cdot \nu _{\varepsilon }(x))({\overline{M}}_\varepsilon (y')\cdot \nu _{\varepsilon }(y))\, \mathrm{d}{{\mathcal {H}}}^{2}(x)\mathrm{d}{{\mathcal {H}}}^2(y)\nonumber \\&= \lim _{\varepsilon \rightarrow 0} \frac{1}{2\pi \varepsilon }\biggl [\int _{\Gamma ^+_\varepsilon }\int _{\Gamma ^+_\varepsilon } \frac{1}{|x-y|}({\overline{M}}_\varepsilon (x')\cdot \nu _{\varepsilon }(x))({\overline{M}}_\varepsilon (y')\cdot \nu _{\varepsilon }(y))\, \mathrm{d}{{\mathcal {H}}}^{2}(x)\mathrm{d}{{\mathcal {H}}}^2(y)\nonumber \\&\qquad +\int _{\Gamma ^+_\varepsilon }\int _{\Gamma ^-_\varepsilon } \frac{1}{|x-y|}({\overline{M}}_\varepsilon (x')\cdot \nu _{\varepsilon }(x))({\overline{M}}_\varepsilon (y')\cdot \nu _{\varepsilon }(y))\, \mathrm{d}{{\mathcal {H}}}^{2}(x)\mathrm{d}{{\mathcal {H}}}^2(y)\biggr ]\nonumber \\&= \lim _{\varepsilon \rightarrow 0} \int _\omega \int _\omega \Gamma _\varepsilon (x',y'){\overline{M}}_\varepsilon (x')\cdot {\overline{M}}_\varepsilon (y') \, \mathrm{d}x'\mathrm{d}y'\,, \end{aligned}$$

where

$$\begin{aligned}&\Gamma _\varepsilon ({ x'},{ y'}) := \\&\frac{1}{2\pi \varepsilon }\left( \frac{n(\frac{{ y'}}{\varepsilon })\otimes n(\frac{{ x'}}{\varepsilon })}{\sqrt{|{x'} -{y'}|^2 + \varepsilon ^2 \left| f\left( \frac{{x'}}{\varepsilon }\right) - f\left( \frac{{ y'}}{\varepsilon }\right) \right| ^2}} \quad -\frac{n(\frac{{ y'}}{\varepsilon })\otimes n(\frac{{ x'}}{\varepsilon })}{\sqrt{|{x'} -{y'}|^2 + \varepsilon ^2 \left| a+ f\left( \frac{{ x'}}{\varepsilon }\right) - f\left( \frac{{ y'}}{\varepsilon }\right) \right| ^2}}\right) , \end{aligned}$$

with

$$\begin{aligned} n(x'):=\left( -\nabla f(x'), 1\right) \,. \end{aligned}$$

Observe now that there exists *L* sufficiently large such that

$$\begin{aligned} |\Gamma _\varepsilon ({ x'}, { y'})| \le \frac{L}{2\pi \varepsilon }\left( \frac{1}{{|{ x'} -{' y}| }} - \frac{1}{\sqrt{|{x'} -{y'}|^2 + \varepsilon ^2 L^2}}\right) =: \frac{L}{2\pi \varepsilon } K_\varepsilon ({x'} - { y'}) \end{aligned}$$

and note that, using also (3.6), we have

3.13 $$\begin{aligned}&\frac{L}{2\pi \varepsilon }\int _{{\mathbb {R}}^2} K_\varepsilon (z')\, \mathrm{d}z' \le \frac{\pi }{2} L^2 \quad \hbox { and for any fixed } \delta>0 \nonumber \\&\quad \frac{L}{2\pi \varepsilon } \int _{|{ z'}| > \delta } K_\varepsilon ({z'})\mathrm{d}z' \rightarrow 0 \hbox { as }\varepsilon \rightarrow 0\,. \end{aligned}$$

We define the *Q*-periodic function

$$\begin{aligned} G(x'):= \frac{1}{2\pi }\int _{{\mathbb {R}}^2}&\Biggl [\frac{n(x')\otimes n({ z'}+ x' )}{\sqrt{|{z'}|^2 + \left| f\left( z'+ x'\right) - f\left( x'\right) \right| ^2}} \\&\quad -\frac{n(x')\otimes n({ z'}+ x' )}{\sqrt{|{z'}|^2 + \left| a+ f\left( z'+ x'\right) - f\left( x'\right) \right| ^2}}\Biggr ] \, \mathrm{d}{ z'}\,. \end{aligned}$$

By the change of variables $z':=(x'-y')/\varepsilon$, we obtain

$$\begin{aligned} G\left( \frac{{ y'}}{\varepsilon } \right) = \int _{{\mathbb {R}}^2} \Gamma _\varepsilon (x', y')\, \mathrm{d}x'. \end{aligned}$$

Thus,

$$\begin{aligned}&\Biggl |\int _{\omega } \Gamma _\varepsilon ({ x'}, { y'}){\overline{M}}_\varepsilon ({ x'}) \, \mathrm{d}x' - G\left( \frac{{ y'}}{\varepsilon } \right) m_0({ y'})\Biggr |\\&\quad = \Biggl |\int _{{\mathbb {R}}^2} \Gamma _\varepsilon ({ x'}, { y'})({\overline{M}}_\varepsilon ({ x'})\chi _\omega (x')- m_0 ({ y'})) \, \mathrm{d}x'\Biggr | \\&\quad \le \frac{L}{2\pi \varepsilon } \int _{{\mathbb {R}}^2} K_\varepsilon ({ x'} - {y'}) |{\overline{M}}_\varepsilon ({x'})\chi _\omega (x') - m_0 ({y'})| \, \mathrm{d}x' \end{aligned}$$

so that

$$\begin{aligned}&\int _{\omega }\Biggl |\int _{\omega } \Gamma _\varepsilon ({ x'}, { y'}){\overline{M}}_\varepsilon ({ x'}) \, \mathrm{d}x' - G\left( \frac{{ y'}}{\varepsilon } \right) m_0({ y'})\Biggr | \mathrm{d}y' \\&\quad \le \frac{L}{2\pi \varepsilon } \int _{{\mathbb {R}}^2}\int _{{\mathbb {R}}^2} K_\varepsilon ({ x'} - {y'}) |{\overline{M}}_\varepsilon ({x'})\chi _\omega (x') - m_0 ({y'})\chi _\omega (y')| \, \mathrm{d}x'\mathrm{d}y'\rightarrow 0\,, \end{aligned}$$

where the last limit follows from Lemma 3.8. In turn, using $|{\overline{M}}_{\varepsilon }(y')| \le 1$ we have

$$\begin{aligned}&\lim _{\varepsilon \rightarrow 0} \int _\omega \int _\omega \Gamma _\varepsilon (x',y') {\overline{M}}_\varepsilon (x')\cdot {\overline{M}}_\varepsilon (y') \, \mathrm{d}x'\mathrm{d}y'\\&\quad = \lim _{\varepsilon \rightarrow 0}\int _\omega G\left( \frac{{ y'}}{\varepsilon } \right) m_0({ y'})\cdot {\overline{M}}_\varepsilon (y') \mathrm{d}y'= \int _\omega A_{\mathrm {hom}}\, m_0\cdot m_0\, \mathrm{d}x'\,, \end{aligned}$$

where the last equality follows from the Riemann–Lebesgue lemma and the definition of *G* and $A_{\mathrm {hom}}$. The conclusion of the lemma follows recalling (3.12). $\square$

Combining Lemma 3.1, Lemmas 3.4–3.7 and Proposition 3.9, we easily establish the following asymptotic behavior of the magnetostatic energy.

#### Proposition 3.10

Let $m_0\in H^1(\omega ; {\mathbb {S}}^2)$ and let ${\overline{M}}_\varepsilon \rightharpoonup m_0$ weakly in $H^1(\omega ; \overline{B(0,1)})$. For every $\varepsilon >0$ let ${\bar{u}}_\varepsilon$ solve (2.3) with *M* replaced by ${\overline{M}}_\varepsilon$. Then

$$\begin{aligned} \frac{1}{\varepsilon }\int _{{\mathbb {R}}^3}|\nabla {\bar{u}}_\varepsilon |^2\, \mathrm{d}x\rightarrow \int _\omega A_{\mathrm {hom}}\, m_0\cdot m_0\, \mathrm{d}x'\,, \end{aligned}$$

as $\varepsilon \rightarrow 0^+$, where $A_{\mathrm {hom}}$ is the matrix defined in (2.9).

### Study of the Magnetostatic Energy: The General Case

In this section, we study the magnetostatic energy in general domains of the form (2.1). We note that Lemmas 3.2–3.7 can be directly transferred to the case of general profiles $f_1$, $f_2$ and therefore, we will be referring to them without loss of generality. As in the previous section, the core of the analysis is represented by the study of the leading order contribution of the magnetostatic energy performed in Proposition 3.13. We notice here that because of the general form of $f_1$ and $f_2$ some of the cancellations we benefitted from in Proposition 3.9 do not occur anymore. This explains the presence of additional terms in the limit and makes the analysis much more involved.

#### Lemma 3.11

Let ${\overline{M}}'_\varepsilon \rightarrow m'_0$ in $L^2 (\omega ; {\mathbb {R}}^2)$, with $|{\overline{M}}'_\varepsilon |\le 1$. Then

$$\begin{aligned} \frac{1}{\varepsilon }\int _{\omega }\int _{\partial \omega }\int _{\varepsilon f_1(x'/\varepsilon )}^{ \varepsilon f_2(x'/\varepsilon )}\frac{({\overline{M}}'_\varepsilon (x')\cdot \nu _{\omega }(x'))({\overline{M}}'_\varepsilon (y')\cdot \nabla f_i(y'/\varepsilon ))}{\sqrt{|x'-y'|^2+(x_3- \varepsilon f_i(y'/\varepsilon ))^2}}\mathrm{d}x_3\mathrm{d}{{\mathcal {H}}}^1(x')\mathrm{d}y'\rightarrow 0 \end{aligned}$$

for $i=1, 2$. Here $\nu _\omega$ denotes the outer unit normal to $\partial \omega$.

#### Proof

Using a change of variable and interchanging integrals, we may rewrite the above integral as

$$\begin{aligned} \int _{\partial \omega }\int _{f_1(x'/\varepsilon )}^{ f_2(x'/\varepsilon )}({\overline{M}}'_\varepsilon (x')\cdot \nu _{\omega }(x'))\int _{\omega }\frac{{\overline{M}}'_\varepsilon (y')\cdot \nabla f_i(y'/\varepsilon )}{\sqrt{|x'-y'|^2+\varepsilon ^2(x_3- f_i(y' /\varepsilon ))^2}}\mathrm{d}y'\mathrm{d}x_3\mathrm{d}{{\mathcal {H}}}^1(x') \end{aligned}$$

Since for all $x=(x', x_3)$

$$\begin{aligned} \frac{{\overline{M}}'_\varepsilon }{\sqrt{|x'- \cdot |^2+\varepsilon ^2(x_3- f_i(\cdot /\varepsilon ))^2}}\rightarrow \frac{m'_0}{|x'- \cdot |}\qquad \text {in }L^1(\omega ; {\mathbb {R}}^2) \end{aligned}$$

and $\nabla f_i(\cdot /\varepsilon ){\mathop {\rightharpoonup }\limits ^{*}} 0$ weakly-$*$ in $L^\infty (\omega ; {\mathbb {R}}^2)$ (due to the periodicity of $f_i$), we deduce that

$$\begin{aligned} \int _{\omega }\frac{{\overline{M}}'_\varepsilon (y')\cdot \nabla f_i(y'/\varepsilon )}{\sqrt{|x'-y'|^2+\varepsilon ^2(x_3- f_i(y'/\varepsilon ))^2}}\mathrm{d}y'\rightarrow 0\qquad \text {for all } x\,. \end{aligned}$$

Since the above integral is uniformly bounded with respect to *x*, the thesis of the lemma follows by the dominated convergence theorem. $\square$

As a consequence of the previous lemma, we may now show the following

#### Lemma 3.12

Let ${\overline{M}}_\varepsilon =({\overline{M}}'_\varepsilon , {\overline{M}}^3_\varepsilon )\rightarrow m_0=(m'_0, m^3_0)$ in $L^2 (\omega ; {\mathbb {R}}^3)$, with $|{\overline{M}}_\varepsilon |\le 1$. Then

$$\begin{aligned}&\frac{1}{\varepsilon }\int _{\omega }\int _{\partial \omega }\int _{\varepsilon f_1(x'/\varepsilon )}^{ \varepsilon f_2(x'/\varepsilon )}\frac{({\overline{M}}'_\varepsilon (x')\cdot \nu _{\omega }(x'))({\overline{M}}_\varepsilon (y')\cdot n_2(y'/\varepsilon ))}{\sqrt{|x'-y'|^2+(x_3- \varepsilon f_2(y'/\varepsilon ))^2}}\mathrm{d}x_3\mathrm{d}{{\mathcal {H}}}^1(x')\mathrm{d}y'\\&\quad -\frac{1}{\varepsilon }\int _{\omega }\int _{\partial \omega }\int _{\varepsilon f_1(x'/\varepsilon )}^{ \varepsilon f_2(x'/\varepsilon )}\frac{({\overline{M}}'_\varepsilon (x')\cdot \nu _{\omega }(x'))({\overline{M}}_\varepsilon (y')\cdot n_1(y'/\varepsilon ))}{\sqrt{|x'-y'|^2+(x_3- \varepsilon f_1(y'/\varepsilon ))^2}}\mathrm{d}x_3\mathrm{d}{{\mathcal {H}}}^1(x')\mathrm{d}y'\rightarrow 0\,. \end{aligned}$$

Here $n_1$ and $n_2$ are the vectors defined in (2.8).

#### Proof

Observe that the difference of the two integrals appearing in the statement can be rewritten as

$$\begin{aligned}&-\frac{1}{\varepsilon }\int _{\omega }\int _{\partial \omega }\int _{\varepsilon f_1(x'/\varepsilon )}^{ \varepsilon f_2(x'/\varepsilon )}\frac{({\overline{M}}'_\varepsilon (x')\cdot \nu _{\omega }(x'))({\overline{M}}'_\varepsilon (y')\cdot \nabla f_2(y'/\varepsilon ))}{\sqrt{|x'-y'|^2+(x_3- \varepsilon f_2(y'/\varepsilon ))^2}}\mathrm{d}x_3\mathrm{d}{{\mathcal {H}}}^1(x')\mathrm{d}y' \\&\quad +\frac{1}{\varepsilon }\int _{\omega }\int _{\partial \omega }\int _{\varepsilon f_1(x'/\varepsilon )}^{ \varepsilon f_2(x'/\varepsilon )}\frac{({\overline{M}}'_\varepsilon (x')\cdot \nu _{\omega }(x'))({\overline{M}}'_\varepsilon (y')\cdot \nabla f_1(y'/\varepsilon ))}{\sqrt{|x'-y'|^2+(x_3- \varepsilon f_1(y'/\varepsilon ))^2}}\mathrm{d}x_3\mathrm{d}{{\mathcal {H}}}^1(x')\mathrm{d}y'\\&\quad + \frac{1}{\varepsilon }\int _{\omega }\int _{\partial \omega }\int _{\varepsilon f_1(x'/\varepsilon )}^{ \varepsilon f_2(x'/\varepsilon )}{\overline{M}}^3_\varepsilon (y') ({\overline{M}}'_\varepsilon (x')\cdot \nu _{\omega }(x')) \Biggl (\frac{1}{\sqrt{|x'-y'|^2+(x_3- \varepsilon f_2(y'/\varepsilon ))^2}}\\&- \frac{1}{\sqrt{|x'-y'|^2+(x_3- \varepsilon f_1(y'/\varepsilon ))^2}}\Biggl ) \mathrm{d}x_3\mathrm{d}{{\mathcal {H}}}^1(x')\mathrm{d}y'\,. \end{aligned}$$

Now, the first two integrals in the above formula vanish thanks to Lemma 3.11, while the convergence to zero of the last one can be shown as in Lemma 3.6. $\square$

We are ready to prove the main result, which establishes the limiting behavior of the magnetostatic energy.

#### Proposition 3.13

Let ${\overline{M}}_\varepsilon \rightharpoonup m_0$ weakly in $H^1(\omega ; {\mathbb {S}}^2)$. Then

$$\begin{aligned}&\frac{1}{4\pi \varepsilon }\int _{\partial V_\varepsilon }\int _{\partial V_\varepsilon } \frac{1}{|x-y|}({\overline{M}}_\varepsilon (x')\cdot \nu _{{\varepsilon }}(x))( {\overline{M}}_\varepsilon (y')\cdot \nu _{{\varepsilon }}(y))\, \mathrm{d}{{\mathcal {H}}}^{2}(x)\mathrm{d}{{\mathcal {H}}}^2(y)\\&\quad \rightarrow \int _\omega A_{\mathrm {hom}} m_0\cdot m_0\, \mathrm{d}x'\,, \end{aligned}$$

with $A_{\mathrm {hom}}$ defined in (2.7). We recall that $\nu _\varepsilon$ stands for the outer unit normal to $\partial V_\varepsilon$.

#### Proof

We start by decomposing the double integral $\int _{\partial V_\varepsilon }\int _{\partial V_\varepsilon }$ similarly to (3.9) and observing that by Lemmas 3.12 and 3.7 the terms involving lateral boundary $\partial \omega$ vanish in the limit as $\varepsilon \rightarrow 0$. Therefore, we have

3.14 $$\begin{aligned}&\lim _{\varepsilon \rightarrow 0} \frac{1}{4\pi \varepsilon }\int _{\partial V_\varepsilon }\int _{\partial V_\varepsilon } \frac{1}{|x-y|}({\overline{M}}_\varepsilon (x')\cdot \nu _{\varepsilon }(x))( {\overline{M}}_\varepsilon (y')\cdot \nu _{\varepsilon }(y))\, \mathrm{d}{{\mathcal {H}}}^{2}(x)\mathrm{d}{{\mathcal {H}}}^2(y) \nonumber \\&= \lim _{\varepsilon \rightarrow 0} \Biggl ( \frac{1}{4\pi \varepsilon }\int _{\omega }\int _{\omega } \frac{({\overline{M}}_\varepsilon (x')\cdot n_1(x'/\varepsilon ))({\overline{M}}_\varepsilon (y')\cdot n_1(y'/\varepsilon ))}{\sqrt{|x'-y'|^2+\varepsilon ^2(f_1(x'/\varepsilon )-f_1(y'/\varepsilon ))^2}}\mathrm{d}x'\mathrm{d}y'\nonumber \\&\quad +\frac{1}{4\pi \varepsilon }\int _{\omega }\int _{\omega } \frac{({\overline{M}}_\varepsilon (x')\cdot n_2(x'/\varepsilon ))({\overline{M}}_\varepsilon (y')\cdot n_2(y'/\varepsilon ))}{\sqrt{|x'-y'|^2+\varepsilon ^2(f_2(x'/\varepsilon )-f_2(y'/\varepsilon ))^2}}\mathrm{d}x'\mathrm{d}y'\nonumber \\&\quad -\frac{1}{2\pi \varepsilon }\int _{\omega }\int _{\omega } \frac{({\overline{M}}_\varepsilon (x')\cdot n_1(x'/\varepsilon ))({\overline{M}}_\varepsilon (y')\cdot n_2(y'/\varepsilon ))}{\sqrt{|x'-y'|^2+\varepsilon ^2(f_1(x'/\varepsilon )-f_2(y'/\varepsilon ))^2}}\mathrm{d}x'\mathrm{d}y' \Biggr ) \end{aligned}$$

3.15 $$\begin{aligned}&=: \lim _{\varepsilon \rightarrow 0}I_\varepsilon \,. \end{aligned}$$

Now, notice that

3.16 $$\begin{aligned}&I_\varepsilon = I_\varepsilon \nonumber \\&\pm \frac{1}{4\pi \varepsilon } \int _{\omega }\int _{\omega }\nonumber \\&\quad \frac{({\overline{M}}_\varepsilon (x')\cdot n_2(x'/\varepsilon )-{\overline{M}}_\varepsilon (x')\cdot n_1(x'/\varepsilon ))({\overline{M}}_\varepsilon (y')\cdot n_2(y'/\varepsilon )-{\overline{M}}_\varepsilon (y')\cdot n_1(y'/\varepsilon ))}{\sqrt{|x'-y'|^2+\varepsilon ^2}}\mathrm{d}x'\mathrm{d}y' \nonumber \\&\quad = \frac{1}{4\pi \varepsilon }\int _{\omega }\int _{\omega }({\overline{M}}_\varepsilon (x')\cdot n_1(x'/\varepsilon ))({\overline{M}}_\varepsilon (y')\cdot n_1(y'/\varepsilon ))\nonumber \\&\quad \Biggl (\frac{1}{\sqrt{|x'-y'|^2+\varepsilon ^2(f_1(x'/\varepsilon )-f_1(y'/\varepsilon ))^2}} -\frac{1}{\sqrt{|x'-y'|^2+\varepsilon ^2}}\Biggr )\mathrm{d}x'\mathrm{d}y'\nonumber \\&\quad +\frac{1}{4\pi \varepsilon }\int _{\omega }\int _{\omega }({\overline{M}}_\varepsilon (x')\cdot n_2(x'/\varepsilon ))({\overline{M}}_\varepsilon (y')\cdot n_2(y'/\varepsilon ))\nonumber \\&\quad \Biggl (\frac{1}{\sqrt{|x'-y'|^2+\varepsilon ^2(f_2(x'/\varepsilon )-f_2(y'/\varepsilon ))^2}} -\frac{1}{\sqrt{|x'-y'|^2+\varepsilon ^2}}\Biggr )\mathrm{d}x'\mathrm{d}y'\nonumber \\&\quad - \frac{1}{2\pi \varepsilon }\int _{\omega }\int _{\omega }({\overline{M}}_\varepsilon (x')\cdot n_1(x'/\varepsilon ))({\overline{M}}_\varepsilon (y')\cdot n_2(y'/\varepsilon ))\nonumber \\&\quad \Biggl (\frac{1}{\sqrt{|x'-y'|^2+\varepsilon ^2(f_1(x'/\varepsilon )-f_2(y'/\varepsilon ))^2}} -\frac{1}{\sqrt{|x'-y'|^2+\varepsilon ^2}}\Biggr )\mathrm{d}x'\mathrm{d}y'\nonumber \\&\quad + \frac{1}{4\pi \varepsilon } \int _{\omega }\int _{\omega }\frac{({\overline{M}}'_\varepsilon (x')\cdot \nabla (f_2-f_1)(x'/\varepsilon )) ({\overline{M}}'_\varepsilon (y')\cdot \nabla (f_2-f_1)(y'/\varepsilon ))}{\sqrt{|x'-y'|^2+\varepsilon ^2}}\mathrm{d}x'\mathrm{d}y'\nonumber \\&\quad =:I^1_\varepsilon +I^2_\varepsilon +I^3_\varepsilon +I^4_\varepsilon \,. \end{aligned}$$

Here we used again the notation ${\overline{M}}_\varepsilon =({\overline{M}}'_\varepsilon , M^3_\varepsilon )$. The limits of $I^1_\varepsilon$, $I^2_\varepsilon$ and $I^3_\varepsilon$ can be computed arguing exactly as in the proof of Lemma 3.9. We obtain

3.17 $$\begin{aligned}&I^1_\varepsilon \rightarrow \int _\omega A_{\mathrm {hom},1} m_0\cdot m_0\, \mathrm{d}x'\,, \ I^2_\varepsilon \rightarrow \int _\omega A_{\mathrm {hom},2} m_0\cdot m_0\, \mathrm{d}x'\,, \ \text {and}\ I^3_\varepsilon \nonumber \\&\quad \rightarrow \int _\omega A_{\mathrm {hom},3} m_0\cdot m_0\, \mathrm{d}x'\,, \end{aligned}$$

where

$$\begin{aligned} A_{\mathrm {hom},1}:=&\frac{1}{4\pi }\int _{Q}\int _{{\mathbb {R}}^2}\Biggl ( \frac{n_1(x') \otimes n_1(z'+x') }{\sqrt{|z'|^2 + \left| f_1( z'+x') - f_1(x') \right| ^2}} - \frac{n_1(x') \otimes n_1(z'+x') }{\sqrt{|z'|^2 +1}} \Biggr )\, \mathrm{d}z'\mathrm{d}x' \nonumber \\ A_{\mathrm {hom},2}:=&\frac{1}{4\pi }\int _{Q}\int _{{\mathbb {R}}^2}\Biggl ( \frac{n_2(x') \otimes n_2(z'+x') }{\sqrt{|z'|^2 + \left| f_2( z'+x') - f_2(x') \right| ^2}} - \frac{n_2(x') \otimes n_2(z'+x') }{\sqrt{|z'|^2 +1}} \Biggr )\, \mathrm{d}z'\mathrm{d}x' \nonumber \\ A_{\mathrm {hom},3}:=&- \frac{1}{2\pi }\int _{Q}\int _{{\mathbb {R}}^2}\Biggl ( \frac{n_1(x') \otimes n_2(z'+x') }{\sqrt{|z'|^2 + \left| f_2( z'+x') - f_1(x') \right| ^2}} - \frac{n_1(x') \otimes n_2(z'+x') }{\sqrt{|z'|^2 +1}} \Biggr )\, \mathrm{d}z'\mathrm{d}x'\,. \end{aligned}$$

We are left with studying the behavior of $I^4_\varepsilon$. In order to deal with such a term, we set $g:=f_2-f_1$ and we note that integration by parts yields

3.18 $$\begin{aligned} 4\pi I^4_\varepsilon =&\, \varepsilon \int _{\omega }\int _{\omega }\text{ div }\,_{y'}\biggl [{\overline{M}}'_\varepsilon (y')\text{ div }\,_{x'}\biggl (\frac{{\overline{M}}'_\varepsilon (x')}{\sqrt{|x'-y'|^2+\varepsilon ^2}}\biggr )\biggr ]g(x'/\varepsilon )g(y'/\varepsilon )\mathrm{d}x'\mathrm{d}y'\nonumber \\&+\int _{\partial \omega }({\overline{M}}'_\varepsilon \cdot \nu _{\omega })(x')g(x'/\varepsilon )\int _{\omega }\frac{{\overline{M}}'_\varepsilon (y')\cdot \nabla g(y'/\varepsilon )}{\sqrt{|x'-y'|^2+\varepsilon ^2}}\, \mathrm{d}y' \mathrm{d}{{\mathcal {H}}}^1(x')\nonumber \\&-\varepsilon \int _{\partial \omega }\int _{\omega }({\overline{M}}'_\varepsilon \cdot \nu _{\omega })(y')\text{ div }\,_{x'}\biggl (\frac{{\overline{M}}'_\varepsilon (x')}{\sqrt{|x'-y'|^2+\varepsilon ^2}}\biggr )g(x'/\varepsilon )g(y'/\varepsilon )\,\mathrm{d}y' \mathrm{d}{{\mathcal {H}}}^1(x')\nonumber \\ =:&J^1_\varepsilon +J^2_\varepsilon +J^3_\varepsilon \,. \end{aligned}$$

Arguing exactly as in the proof of Lemma 3.11, the $L^{\infty }$ weak-$*$ convergence to 0 of $\nabla g(\cdot /\varepsilon )$ easily yields that

3.19 $$\begin{aligned} J^2_\varepsilon \rightarrow 0\,. \end{aligned}$$

Moreover, for a sufficiently large $C>0$, we have

3.20 $$\begin{aligned} |J^3_\varepsilon |&\le \varepsilon \Vert g_{\infty }\Vert _\infty ^2 \left( \int _{\partial \omega }\int _{\omega }\frac{|\text{ div }\,_{x'}{\overline{M}}'_\varepsilon |}{\sqrt{|x'-y'|^2+\varepsilon ^2}}\, \mathrm{d}x'\mathrm{d}{{\mathcal {H}}}^1(y') + \int _{\partial \omega }\int _{\omega }\frac{|x'-y'|}{(|x'-y'|^2+\varepsilon ^2)^{3/2}}\, \mathrm{d}x'\mathrm{d}{{\mathcal {H}}}^1(y') \right) \nonumber \\&\le \sqrt{\varepsilon } \Vert g_{\infty }\Vert _\infty ^2 \int _{\partial \omega }\int _{\omega }\frac{|\text{ div }\,_{x'}{\overline{M}}'_\varepsilon |}{\sqrt{|x'-y'|}}\, \mathrm{d}x'\mathrm{d}{{\mathcal {H}}}^1(y') +C\varepsilon \int _0^C\frac{r^2}{(r^2+\varepsilon ^2)^{3/2}}\, \mathrm{d}r\nonumber \\&\le C\sqrt{\varepsilon }+ C\varepsilon \int _0^C\frac{r}{(r^2+\varepsilon ^2)}\, \mathrm{d}r\rightarrow 0\,, \end{aligned}$$

where the last convergence follows by explicit computation of the integral. Note that in the last inequality we have also used the fact that $\text{ div }\,_{x'}{\overline{M}}'_\varepsilon$ is bounded in $L^2$. In order to deal with $J^1_\varepsilon$, we expand the double divergence term to get

$$\begin{aligned} J^1_\varepsilon&= \varepsilon \int _{\omega }\int _{\omega }\frac{\text{ div }\,_{x'}{\overline{M}}'_\varepsilon (x')\text{ div }\,_{y'}{\overline{M}}'_\varepsilon (y')}{\sqrt{|x'-y'|^2+\varepsilon ^2}}g(x'/\varepsilon )g(y'/\varepsilon )\mathrm{d}x'\mathrm{d}y' \\&\quad +2\varepsilon \int _{\omega }\int _{\omega }\frac{\text{ div }\,_{x'}{\overline{M}}'_\varepsilon (x'){\overline{M}}'_\varepsilon (y')\cdot (x'-y')}{(|x'-y'|^2+\varepsilon ^2)^{3/2}}g(x'/\varepsilon )g(y'/\varepsilon )\mathrm{d}x'\mathrm{d}y'\\&\quad +\varepsilon \int _{\omega }\int _{\omega }\frac{{\overline{M}}'_\varepsilon (x')\cdot {\overline{M}}'_\varepsilon (y')}{(|x'-y'|^2+\varepsilon ^2)^{3/2}}g(x'/\varepsilon )g(y'/\varepsilon )\mathrm{d}x'\mathrm{d}y'\\&\quad -3\varepsilon \int _{\omega }\int _{\omega }\frac{[{\overline{M}}'_\varepsilon (x')\cdot (x'-y')] [{\overline{M}}'_\varepsilon (y')\cdot (x'-y')]}{(|x'-y'|^2+\varepsilon ^2)^{5/2}}g(x'/\varepsilon )g(y'/\varepsilon )\mathrm{d}x'\mathrm{d}y' \\&=: J^{1,1}_\varepsilon +J^{1,2}_\varepsilon +J^{1,3}_\varepsilon +J^{1,4}_\varepsilon \,. \end{aligned}$$

Note that

$$\begin{aligned} J^{1,1}_\varepsilon \le \Vert g\Vert _\infty ^2 \int _{\omega }\int _{\omega }K_\varepsilon (x'-y')|\text{ div }\,_{x'}{\overline{M}}'_\varepsilon (x')|\,|\text{ div }\,_{y'}{\overline{M}}'_\varepsilon (y')|\, \mathrm{d}x'\mathrm{d}y'\,, \end{aligned}$$

where we set

$$\begin{aligned} K_\varepsilon (z'):=\frac{\varepsilon }{\sqrt{|z'|^2+\varepsilon ^2}}\,. \end{aligned}$$

Using the fact that $\Vert K_\varepsilon \Vert _{L^1(B)} \le C \varepsilon$, where *B* is a sufficiently large ball containing $\omega -\omega$, and that $\text{ div }\,{\overline{M}}_\varepsilon$ is bounded in $L^2$, we deduce from the generalized Young’s inequality that $J^{1,1}_\varepsilon \rightarrow 0$. Analogously,

$$\begin{aligned} J^{1,2}_\varepsilon \le 2\Vert g\Vert _\infty ^2 \int _{\omega }\int _{\omega }K'_\varepsilon (x'-y')|\text{ div }\,_{x'}{\overline{M}}'_\varepsilon (x')|\, \mathrm{d}x'\mathrm{d}y'\,, \end{aligned}$$

with

$$\begin{aligned} K'_\varepsilon (z'):=\frac{\varepsilon |z'|}{(|z'|^2+\varepsilon ^2)^{3/2}}\,. \end{aligned}$$

Since $\Vert K'_\varepsilon \Vert _{L^1(B)}\rightarrow 0$ (see (3.20)), we also have $J^{1,2}_\varepsilon \rightarrow 0$ using generalized Young’s inequality. Thus,

$$\begin{aligned} \lim _{\varepsilon \rightarrow 0} J^1_\varepsilon =\lim _{\varepsilon \rightarrow 0}(J^{1,3}_\varepsilon +J^{1,4}_\varepsilon )\,. \end{aligned}$$

The last limit can be now computed arguing as in the proof of Lemma 3.9 to get

3.21 $$\begin{aligned} \lim _{\varepsilon \rightarrow 0} J^1_\varepsilon =\lim _{\varepsilon \rightarrow 0}(J^{1,3}_\varepsilon +J^{1,4}_\varepsilon )= 4\pi \int _\omega A'_{\mathrm {hom}, 4} m'_0\cdot m'_0 \mathrm{d}x'\,, \end{aligned}$$

with

$$\begin{aligned} A'_{\mathrm {hom}, 4}:= \frac{1}{4\pi }\int _{Q}\int _{{\mathbb {R}}^2}\Biggl (\frac{Id}{(|z'|^2+1)^{3/2}}-\frac{3z \otimes z'}{(|z'|^2+1)^{5/2}}\Biggr )g(z'+x')g(x')\, \mathrm{d}z'\mathrm{d}x'\,. \end{aligned}$$

We reproduce here the argument for the reader’s convenience. First of all, note that we can write

$$\begin{aligned} J^{1,3}_\varepsilon +J^{1,4}_\varepsilon =\int _{\omega }\int _{\omega }{{\hat{\Gamma }}}_\varepsilon (x', y') {\overline{M}}'_\varepsilon (x') {\overline{M}}'_\varepsilon (y')\, \mathrm{d}x'\mathrm{d}y'\,, \end{aligned}$$

where

$$\begin{aligned} {{\hat{\Gamma }}}_\varepsilon (x', y'):= \varepsilon \Biggl (\frac{Id}{(|x'-y'|^2+\varepsilon ^2)^{3/2}}-\frac{3(x'-y') \otimes (x'-y')}{(|x'-y'|^2+\varepsilon ^2)^{5/2}}\Biggr )g(x'/\varepsilon )g(y'/\varepsilon )\,, \end{aligned}$$

and note that

$$\begin{aligned} |\Gamma _\varepsilon (x', y')|\le \varepsilon \Vert g\Vert _\infty ^2 \Biggl |\frac{Id}{(|x'-y'|^2+\varepsilon ^2)^{3/2}}-\frac{3(x'-y') \otimes (x'-y')}{(|x'-y'|^2+\varepsilon ^2)^{5/2}}\Biggr |=:{\hat{K}}_\varepsilon (x'-y')\,, \end{aligned}$$

with ${\hat{K}}_\varepsilon$ satisfying (3.13) (with ${\hat{K}}_\varepsilon$ in place of $\frac{L}{2\pi \varepsilon } K_\varepsilon$). Moreover, a change of variables shows that

$$\begin{aligned} {\hat{G}}\left( \frac{{ y'}}{\varepsilon } \right) = \int _{{\mathbb {R}}^2} {{\hat{\Gamma }}}_\varepsilon (x', y')\, \mathrm{d}x'\,, \end{aligned}$$

where

$$\begin{aligned} {\hat{G}}(x'):= \int _{{\mathbb {R}}^2}\Biggl (\frac{Id}{(|z'|^2+1)^{3/2}}-\frac{3z \otimes z'}{(|z'|^2+1)^{5/2}}\Biggr )g(z'+x')g(x')\, \mathrm{d}z'\,. \end{aligned}$$

We can now proceed as in the last part of the proof of Lemma 3.9 to show that

$$\begin{aligned} \int _{\omega }\Biggl |\int _{\omega } {{\hat{\Gamma }}}_\varepsilon ({ x'}, { y'}) {\overline{M}}'_\varepsilon ({ x'}) \, \mathrm{d}x' - {\hat{G}}\left( \frac{{ y'}}{\varepsilon } \right) m'_0({ y'})\Biggr | \mathrm{d}y'\rightarrow 0 \end{aligned}$$

and, in turn,

$$\begin{aligned}&\lim _{\varepsilon \rightarrow 0} \int _\omega \int _\omega {{\hat{\Gamma }}}_\varepsilon (x',y') {\overline{M}}'_\varepsilon (x')\cdot {\overline{M}}'_\varepsilon (y') \, \mathrm{d}x'\mathrm{d}y'\\&\quad = \lim _{\varepsilon \rightarrow 0}\int _\omega {\hat{G}}\left( \frac{{ y'}}{\varepsilon } \right) m'_0({ y'})\cdot {\overline{M}}'_\varepsilon (y') \mathrm{d}y'=4\pi \int _\omega A'_{\mathrm {hom}, 4} m'_0\cdot m'_0\, \mathrm{d}x'\,. \end{aligned}$$

This establishes (3.21). Collecting (3.17)–(3.21), we conclude the proof of the proposition. $\square$

As at the end of Sect. 3.1, we can combine the previous results to obtain the following:

#### Proposition 3.14

Let $m_0\in H^1(\omega ; {\mathbb {S}}^2)$ and let ${\overline{M}}_\varepsilon \rightharpoonup m_0$ weakly in $H^1(\omega ; \overline{B(0,1)})$. For every $\varepsilon >0$ let ${\bar{u}}_\varepsilon$ solve (2.3) with *M* replaced by ${\overline{M}}_\varepsilon$. Then

$$\begin{aligned} \frac{1}{\varepsilon }\int _{{\mathbb {R}}^3}|\nabla {\bar{u}}_\varepsilon |^2\, \mathrm{d}x\rightarrow \int _\omega A_{\mathrm {hom}}\, m_0\cdot m_0\, \mathrm{d}x'\,, \end{aligned}$$

as $\varepsilon \rightarrow 0^+$, where $A_{\mathrm {hom}}$ is the matrix defined in (2.7).

### Study of the Exchange Energy

In this section, we identify the limiting exchange energy. We start with the following simple extension argument.

#### Lemma 3.15

Let $M>\max \{\Vert f_1\Vert _\infty , \Vert f_2\Vert _\infty \}$ and set $\Omega ^M:=\omega \times (0,M)$. Let $\{m_\varepsilon \}$ be such that $m_\varepsilon \in H^1(\Omega _\varepsilon ; {\mathbb {S}}^2)$ for every $\varepsilon >0$ and

3.22 $$\begin{aligned} \sup _{\varepsilon >0} \int _{\Omega _\varepsilon } \left( | \nabla _{x'} m_\varepsilon |^2 + \frac{1}{\varepsilon ^2} |\partial _{x_3} m_\varepsilon |^2 \right) \, \mathrm{d}x<+\infty . \end{aligned}$$

Then for every $\varepsilon >0$ there exists ${\tilde{m}}_\varepsilon \in H^1(Q_M; {\mathbb {S}}^2)$ such that ${\tilde{m}}_\varepsilon =m_\varepsilon$ in $\Omega _\varepsilon$ and

3.23 $$\begin{aligned} \sup _\varepsilon \int _{\Omega ^M} \left( | \nabla _{x'} {\tilde{m}}_\varepsilon |^2 + \frac{1}{\varepsilon ^2} |\partial _{x_3} {\tilde{m}}_\varepsilon |^2 \right) \, \mathrm{d}x<+\infty . \end{aligned}$$

#### Proof

The required extension is obtained through repeated vertical reflections with respect to the graphs of $f_1$ and $f_2$. More precisely, for every $k\in {\mathbb {N}}$, $k\ge 3$, we set $f_k:=f_2+(k-2)(f_2-f_1)$ and for $k\in {\mathbb {Z}}$, with $k\le 0$, set $f_k:=f_1+ (k-1)(f_2-f_1)$. Moreover, for every $\varepsilon >0$ and $k\in {\mathbb {Z}}$ denote

$$\begin{aligned} \Omega _\varepsilon ^k:=\left\{ (x', x_3):\, x'\in \omega , f_k\left( \frac{x'}{\varepsilon }\right)< x_3<f_{k+1}\left( \frac{x'}{\varepsilon }\right) \right\} \end{aligned}$$

In particular, note that $\Omega _\varepsilon ^1=\Omega _\varepsilon$. Set $m_\varepsilon ^1:=m_\varepsilon$ on $\Omega _\varepsilon$ and inductively define $m_\varepsilon ^k$ on $\Omega _\varepsilon ^k$ as

$$\begin{aligned} m_\varepsilon ^k(x', x_3):={\left\{ \begin{array}{ll} m_\varepsilon ^{k-1}\left( x', 2 f_k\left( \frac{x'}{\varepsilon }\right) -x_3\right) \qquad \text {if }k\ge 2\,, \\ m_\varepsilon ^{k+1}\left( x', 2 f_{k+1}\left( \frac{x'}{\varepsilon }\right) -x_3\right) \quad \text {if }k\le 0\,. \end{array}\right. } \end{aligned}$$

Finally, we let ${\tilde{m}}_\varepsilon : \omega \times {\mathbb {R}}\rightarrow {\mathbb {S}}^2$ be defined as ${\tilde{m}}_\varepsilon :=m_\varepsilon ^k$ on $\Omega _\varepsilon ^k$. In order to proof (3.23), it clearly suffices to show that for every $k\in {\mathbb {Z}}$ we have

3.24 $$\begin{aligned} \sup _\varepsilon \int _{\Omega ^k_\varepsilon } \left( | \nabla _{x'} m^k_\varepsilon |^2 + \frac{1}{\varepsilon ^2} |\partial _{x_3} m^k_\varepsilon |^2 \right) \, \mathrm{d}x<+\infty \,. \end{aligned}$$

To this aim, observe that for $k\ge 2$ we have

$$\begin{aligned}&\nabla m_\varepsilon ^k(x', x_3)=\biggl (\nabla _{x'}m_\varepsilon ^{k-1}\Bigl (x', 2 f_k\Bigl (\frac{x'}{\varepsilon }\Bigr )-x_3\Bigr )\\&\quad +\frac{2}{\varepsilon }\partial _{x_3}m_\varepsilon ^{k-1}\Bigl (x', 2 f_k\Bigl (\frac{x'}{\varepsilon }\Bigr )-x_3\Bigr )\nabla f_k\Bigl (\frac{x'}{\varepsilon }\Bigr ),\\&\quad -\partial _{x_3}m_\varepsilon ^{k-1}\Bigl (x', 2 f_k\Bigl (\frac{x'}{\varepsilon }\Bigr )-x_3\Bigr )\biggr )\,. \end{aligned}$$

Thus, (3.24) follows easily by induction for $k\ge 2$ recalling that by (3.22) we have

$$\begin{aligned} \sup _\varepsilon \left\| \Bigl (\nabla _{x'} m^1_\varepsilon , \frac{1}{\varepsilon }\partial _{x_3} m_\varepsilon ^1\Bigr )\right\| _{L^2(\Omega _\varepsilon ^1; {\mathbb {M}}^{3\times 3})}<+\infty \,. \end{aligned}$$

The proof for $k\le 0$ is analogous. $\square$

We are now ready to proof the $\Gamma$-liminf inequality for the exchange energy.

#### Proposition 3.16

Let $m_0\in H^1(\omega ; {\mathbb {S}}^2)$ and let $\{m_\varepsilon \}_\varepsilon$ be such that $m_\varepsilon \in H^1(\Omega _\varepsilon ; {\mathbb {S}}^2)$ for every $\varepsilon >0$ and

3.25 $$\begin{aligned} \int _{\Omega _\varepsilon }|m_\varepsilon (x)-m_0(x')|^2\, \mathrm{d}x\rightarrow 0 \end{aligned}$$

as $$\varepsilon \rightarrow 0^+$$. Then

3.26 $$\begin{aligned} \int _{\omega }g_{\mathrm {hom}}(\nabla _{x'} m_0)\, \mathrm{d}x'\le \liminf _{\varepsilon \rightarrow 0} \int _{\Omega _\varepsilon } \left( | \nabla _{x'} m_\varepsilon |^2 + \frac{1}{\varepsilon ^2} |\partial _{x_3} m_\varepsilon |^2 \right) \, \mathrm{d}x\,, \end{aligned}$$

where $g_{\mathrm {hom}}$ is the homogenized exchange energy density defined in (2.6).

When $f_2=-f_1+a$ for some $a>0$, the above result is proven in Braides et al. (2000). It is also clear that the methods of Braides et al. (2000) could be adapted to deal with thin films of the form (2.1). However, for the reader’s convenience we prefer to give here a simple self-contained proof based on the two-scale approach developed in Neukamm (2010). Following Neukamm (2010) (see also Neukamm and Velčić 2013), we consider the following notion of two-scale convergence adapted to the 3D–2D dimension reduction framework with the purpose of capturing the in-plane oscillations.

#### Definition 3.17

Let $\Omega ^M$ be as in Lemma 3.15, let *H* be a finite-dimensional Hilbert space, and let $\{g_\varepsilon \}\subset L^2(\Omega ^M; H)$ be $L^2$-bounded. For any subsequence $\varepsilon _n\searrow 0$, we say that $\{g_{\varepsilon _n}\}$ two-scale converges to *g*, with $g\in L^2\left( \Omega ^M; L^2(Q; H)\right)$, and we write $g_{\varepsilon _n} {\mathop {\rightharpoonup }\limits ^{2\text {-s}}} g$, if

$$\begin{aligned} \lim _{n }\int _{\Omega ^M}\Bigl \langle g_{\varepsilon _n}(x), \psi \Bigl (x,\frac{x'}{\varepsilon _n}\Bigr )\Bigr \rangle \, \mathrm{d}x=\int _{\Omega ^M}\int _{Q}\langle g(x, y'), \psi (x, y')\rangle \, \mathrm{d}y'\, \mathrm{d}x \end{aligned}$$

for all $\psi \in L^2(\Omega ^M; C_\#(Q; H))$. Here, $C_\#(Q; H)$ denotes the space of the *Q*-periodic continuous functions from ${\mathbb {R}}^2$ to *H*, endowed with the sup norm on *Q*, and $\langle \cdot , \cdot \rangle$ stands for the scalar product of *H*.

#### Definition 3.18

Any function $\psi \in L^2(\Omega ^M; C_\#(Q; H))$ will be called an *admissible* test function for the two-scale convergence defined in Definition 3.17.

#### Proof of Proposition 3.16

Without loss of generality, we may assume that (3.22) holds. Let $\{{\tilde{m}}_\varepsilon \}$ be the family of extensions provided by Lemma 3.15. In particular, (3.23) holds and ${\tilde{m}}_\varepsilon \rightharpoonup m_0$ weakly in $H^1(\Omega ^M; {\mathbb {S}}^2)$. Fix a subsequence $\varepsilon _n$ along which the liminf in (3.26) is achieved. Thus, denoting by ${\mathcal {Y}}$ the subspace of $H^1((0,M)\times Q; {\mathbb {R}}^3)$ of functions $m=m(x_3, y' )$ that are *Q*-periodic in the $y'$-variable, we may thus apply (Neukamm 2010, Theorem 6.3.3) and find $m_1=m_1(x', x_3, y')\in L^2(\omega ; {\mathcal {Y}})$ and a (not relabeled) subsequence such that

3.27 $$\begin{aligned} \Bigl (\nabla _{x'}{\tilde{m}}_{\varepsilon _n}, \frac{1}{\varepsilon _n}\partial _{x_3}{\tilde{m}}_{\varepsilon _n}\Bigr ){\mathop {\rightharpoonup }\limits ^{2\text {-s}}} (\nabla _{x'}m_0+\nabla _{y'}m_1, \partial _{x_3}m_1 ) \end{aligned}$$

in the sense of Definition 3.17, that is,

$$\begin{aligned}&\lim _n \int _{\Omega ^M}\Bigl \langle \Bigl (\nabla _{x'}{\tilde{m}}_{\varepsilon _n}(x), \frac{1}{\varepsilon _n}\partial _{x_3}{\tilde{m}}_{\varepsilon _n}(x)\Bigr ), \psi \Bigl (x,\frac{x'}{\varepsilon _n}\Bigr )\Bigr \rangle \, \mathrm{d}x \\&\quad =\int _{\Omega ^M}\int _{Q}\Bigl \langle \Big (\nabla _{x'}m_0(x')+\nabla _{y'}m_1(x, y'), \partial _{x_3}m_1(x,y') \Big ), \psi (x, y')\Bigr \rangle \, \mathrm{d}y'\, \mathrm{d}x \end{aligned}$$

for all $\psi \in L^2(\Omega ^M; C_\#(Q; {\mathbb {M}}^{3\times 3}))$. For $\eta >0$ we can define

$$\begin{aligned} m^\eta _1(x',x_3, y'):=\int _{{\mathbb {R}}^2}\rho _\eta (y'- z')m_1(x',x_3, z')\, \mathrm{d}z' \end{aligned}$$

for almost every $(x', x_3)\in \Omega ^M$ and for all $y'\in Q$, where $(\rho _\eta )_\eta$ stands for the standard family of mollifiers on ${\mathbb {R}}^2$. Note that in particular $\nabla _{x_3, y'}m_1^\eta \in L^2(\Omega ^M; C_\#(Q; {\mathbb {M}}^{3\times 3}))$ for every $\eta >0$ and thus it can be used as a test function for the two-scale convergence, see Definition 3.18.

For every $k\in {\mathbb {N}}$, $x_3\in (0,M)$, and $y'\in {\mathbb {R}}^2$ set

$$\begin{aligned} g_k(x_3, y'):=\inf \{\chi _{(f_1(z'), f_2(z'))}(t)+k|(t,z')-(x_3, y')|:\, t\in (0,M),\, z'\in {\mathbb {R}}^2\}\,, \end{aligned}$$

so that $$0\le g_k\le 1$$

3.28 $$\begin{aligned} g_k(x_3, y')\nearrow g(x_3, y'):=\chi _{(f_1(y'), f_2(y'))}(x_3) \end{aligned}$$

as $k\rightarrow \infty$. Note also that by construction $g_k$ is *k*-Lipschitz continuous and *Q*-periodic in the $y'$-variable. Therefore, it is an admissible test function for the two-scale convergence. Notice that for every *n*, $k\in {\mathbb {N}}$ and $\eta >0$ we have

$$\begin{aligned} \int _{\Omega _{\varepsilon _n}} \Bigl ( | \nabla _{x'} m_{\varepsilon _n}|^2&+ \frac{1}{\varepsilon _n^2} |\partial _{x_3} m_{\varepsilon _n}|^2 \Bigr )\, \mathrm{d}x \ge \int _{\Omega ^M}g_k\Bigr (x_3, \frac{x'}{\varepsilon _n}\Bigl ) \left( | \nabla _{x'} {\tilde{m}}_{\varepsilon _n}|^2 + \frac{1}{\varepsilon _n^2} |\partial _{x_3} {\tilde{m}}_{\varepsilon _n}|^2 \right) \, \mathrm{d}x\\&\ge - \int _{\Omega ^M}g_k\Bigr (x_3, \frac{x'}{\varepsilon _n}\Bigl ) \Bigl | \nabla _{x'} m_{0}(x')+\nabla _{y'}m_1^\eta \Bigl (x,\frac{x'}{\varepsilon _n}\Bigr ) \Bigr |^2\, \mathrm{d}x\\&\quad +2\int _{\Omega ^M}g_k\Bigr (x_3, \frac{x'}{\varepsilon _n}\Bigl ) \Bigl \langle \nabla _{x'} m_{0}(x') +\nabla _{y'}m_1^\eta \Bigl (x,\frac{x'}{\varepsilon _n}\Bigr ), \nabla _{x'}{\tilde{m}}_{\varepsilon _n}(x)\Bigr \rangle \, \mathrm{d}x\\&\quad - \int _{\Omega ^M}g_k\Bigr (x_3, \frac{x'}{\varepsilon _n}\Bigl ) \Bigl |\partial _{x_3}m_1^\eta \Bigl (x,\frac{x'}{\varepsilon _n}\Bigr ) \Bigr |^2\, \mathrm{d}x\\&\quad +2\int _{\Omega ^M}g_k\Bigr (x_3, \frac{x'}{\varepsilon _n}\Bigl )\Bigl \langle \frac{1}{\varepsilon _n}\partial _{x_3}{\tilde{m}}_{\varepsilon _n}(x) , \partial _{x_3}m_1^\eta \Bigl (x,\frac{x'}{\varepsilon _n}\Bigr ) \Bigr \rangle \, \mathrm{d}x \end{aligned}$$

Recalling that $g_k\Bigl (\cdot , \frac{\cdot }{\varepsilon _n}\Bigr ){\mathop {\rightharpoonup }\limits ^{2\text {-s}}} g_k$ as $n\rightarrow \infty$, using (3.27) and the admissibility of $\nabla _{x_3, y'}m_1^\eta$, $g_k$ as test functions for the two-scale convergence, we deduce that

$$\begin{aligned}&\liminf _n \int _{\Omega _{\varepsilon _n}} \Bigl ( | \nabla _{x'} m_{\varepsilon _n}|^2 + \frac{1}{\varepsilon _n^2} |\partial _{x_3} m_{\varepsilon _n}|^2 \Bigr )\, \mathrm{d}x\\&\ge \int _{\Omega ^M}\int _Q g_k(x_3, y') \biggl [ -| \nabla _{x'} m_{0}(x')+\nabla _{y'}m_1^\eta (x,y') |^2\\&\quad +2\langle \nabla _{x'} m_{0}(x') +\nabla _{y'}m_1^\eta (x,y'), \nabla _{x'}m_0(x')+\nabla _{y'}m_1(x, y') \rangle \, \\&\quad - |\partial _{x_3}m_1^\eta (x,y') |^2 + 2 \langle \partial _{x_3} m_{1}(x, y') , \partial _{x_3}m_1^\eta (x,y')\rangle \biggr ]\,\mathrm{d}y' \mathrm{d}x\,. \end{aligned}$$

In turn, recalling (3.28) and that $\nabla _{x_3, y'}m_1^\eta \rightarrow \nabla _{x_3, y'}m_1$ in $L^2(\Omega ^M; L^2(Q; {\mathbb {M}}^{3\times 3})$ as $\eta \rightarrow 0^+$, we may conclude

$$\begin{aligned}&\liminf _n \int _{\Omega _{\varepsilon _n}} \Bigl ( | \nabla _{x'} m_{\varepsilon _n}|^2 + \frac{1}{\varepsilon _n^2} |\partial _{x_3} m_{\varepsilon _n}|^2 \Bigr )\, \mathrm{d}x \\&\quad \ge \int _{\Omega ^M}\int _Q g(x_3, y') \Bigl [ | \nabla _{x'} m_{0}(x')+\nabla _{y'}m_1(x,y') |^2 + |\partial _{x_3}m_1(x,y') |^2\Bigr ]\, \mathrm{d}y'\mathrm{d}x\\&\quad = \int _\omega \int _{Q_{f_1,f_2}} \Bigl [ | \nabla _{x'} m_{0}(x')+\nabla _{y'}m_1(x',x_3, y') |^2 + |\partial _{x_3}m_1(x', x_3, ,y') |^2\Bigr ]\, \mathrm{d}y'\mathrm{d}x_3 \mathrm{d}x'\\&\quad \ge \int _{\omega }g_{\mathrm {hom}}(\nabla _{x'} m_0)\, \mathrm{d}x'\,, \end{aligned}$$

where the last inequality follows from the very definition (2.6) of $g_{\mathrm {hom}}$, recalling that for a.e. $x'\in \omega$ we have $m_1(x', \cdot , \cdot )\in H^1_{\#}(Q_{f_1,f_2};\,{\mathbb {R}}^3)$. This concludes the proof of the proposition. $\square$

We now seek to prove the upper bound. We start with the following remark.

#### Remark 3.19

(Cell formula revisited) Since $g_{\mathrm {hom}}$ is defined by minimizing a nonnegative quadratic form on a linear function space, standard arguments show that $g_{\mathrm {hom}}$ is in turn a nonnegative quadratic form, and thus continuous. Moreover, using the periodicity condition in the definition of the function space it is easy to see that $g_{\mathrm {hom}}$ is positive definite. Also, by strict convexity, the minimizer $\varphi _\xi$ of (2.6) is unique up to adding constant vectors. Let now $s\in {\mathbb {S}}^2$ be such that $\xi ^t s=0$ (that is, *s* is orthogonal to both columns of $\xi$). Then, setting $\psi _\xi :=\varphi _\xi - (\varphi _\xi \cdot s)s$ we can argue as in [Alouges and Fratta (2015) page 10] to show that

$$\begin{aligned}&|\xi +\nabla _{y'}\varphi _\xi |^2+|\partial _{y_3}\varphi _\xi |^2\ge |\xi +\nabla _{y'}\psi _\xi |^2+|\partial _{y_3}\psi _\xi |^2+|\nabla (\varphi _\xi \cdot s)|^2\\&\quad \ge |\xi +\nabla _{y'}\psi _\xi |^2+|\partial _{y_3}\psi _\xi |^2\,. \end{aligned}$$

It follows that $\psi _\xi$ is also a solution and thus $\nabla (\varphi _\xi \cdot s)\equiv 0$, that is, $\varphi _\xi \cdot s$ is constant. Therefore, upon adding a suitable constant vector, we may assume that the solution $\varphi _\xi$ to (2.6) satisfies

$$\begin{aligned} {\left\{ \begin{array}{ll} \int _{Q_{f_1, f_2}}\varphi _\xi \, \mathrm{d}x=0\,,\\ \varphi _\xi \cdot s=0 \text { in }Q_{f_1, f_2}\,. \end{array}\right. } \end{aligned}$$

The above conditions determine $\varphi _\xi$ uniquely. Finally, choosing $\varphi =0$ as a test function in (2.6) we immediately get $g_{\mathrm {hom}}(\xi )\le |\xi |^2$ for all $\xi \in {\mathbb {M}}^{3\times 2}$.

#### Lemma 3.20

Let $M>0$ be as in Lemma 3.15 and denote by ${\mathcal {Y}}$ the subspace of $H^1(Q\times (0,M); {\mathbb {R}}^3)$ of functions $m=m(y)$ that are *Q*-periodic in the $y'$-variable. Let $m_0\in C^1({\overline{\omega }}; {\mathbb {S}}^2)$ then, for $g_{\mathrm {hom}}$ defined in (2.6), the following identity holds:

3.29 $$\begin{aligned}&\int _{\omega }g_{\mathrm {hom}}(\nabla _{x'} m_0)\, \mathrm{d}x'\nonumber \\&\quad =\inf \biggl \{ \int _\omega \int _{Q_{f_1,f_2}} \Bigl [ | \nabla _{x'} m_{0}(x')+\nabla _{y'}m(x',y) |^2+ |\partial _{y_3}m(x', y) |^2\Bigr ]\, \mathrm{d}y \mathrm{d}x'\nonumber \\&\qquad :m\in C^1({\overline{\omega }}; {\mathcal {Y}})\text { s.t. } m(x', y)\cdot m_0(x')\nonumber \\&\quad \equiv 0\text { for a.e. }(x', y)\in \omega \times [Q\times (0,M)] \biggr \}\,. \end{aligned}$$

#### Proof

Without loss of generality, we may assume that $m_0\in C^1({\mathbb {R}}^2; {\mathbb {S}}^2)$. Now for every $x'\in {\mathbb {R}}^2$ let ${\overline{m}}(x', \cdot )\in H^1_{\#}(Q_{f_1,f_2};\,{\mathbb {R}}^3)$ be the *unique* solution to

3.30 $$\begin{aligned} {\left\{ \begin{array}{ll} {\overline{m}}(x', \cdot ) \text { solves } 2.6 \text { with }\xi \text { replaced by }\nabla _{x'} m_0(x')\,,\\ \int _{Q_{f_1, f_2}}{\overline{m}}(x', y) \mathrm{d}y=0\,,\\ {\overline{m}}(x', \cdot )\cdot m_0(x')=0 \text { in }Q_{f_1, f_2}\,. \end{array}\right. } \end{aligned}$$

The solution to the above problem exists and is unique, thanks to Remark 3.19, since $m_0$ is ${\mathbb {S}}^2$-valued and thus $\nabla m_0(x')^t m_0(x')=0$ for all $x'$. By repeated reflections of ${\overline{m}}(x', \cdot )$ with respect to the $y_3$-variable (as in the proof of Lemma 3.15), we may in fact assume that ${\overline{m}}(x',\cdot )\in {\mathcal {Y}}$ and that the third equation in (3.30) holds in $Q\times (0,M)$. Due to uniqueness, it is easy to see that ${\overline{m}}\in C^0({\mathbb {R}}^2; {\mathcal {Y}})$. In particular, ${\overline{m}}$ and $\nabla _y{\overline{m}}$ are globally measurable and

3.31 $$\begin{aligned} \int _{\omega }g_{\mathrm {hom}}(\nabla _{x'} m_0)\, \mathrm{d}x'= \int _\omega \int _{Q_{f_1,f_2}} \Bigl [ | \nabla _{x'} m_{0}(x')+\nabla _{y'}{\overline{m}}(x',y) |^2+ |\partial _{y_3}{\overline{m}}(x', y) |^2\Bigr ]\, \mathrm{d}y \mathrm{d}x'.\nonumber \\ \end{aligned}$$

Let $(\rho _\eta )_{\eta >0}$ be a family of standard mollifiers on ${\mathbb {R}}^2$ and for every $y\in Q\times (0,M)$ set ${\overline{m}}_\eta (\cdot , y):=\rho _\eta *{\overline{m}}(\cdot , y)$, that is, ${\overline{m}}_\eta$ is defined by taking the convolution of ${\overline{m}}$ with respect to the $x'$-variable. Note that by the properties of convolutions we have ${\overline{m}}_\eta \in C^\infty ({\mathbb {R}}^2; {\mathcal {Y}})$ and ${\overline{m}}_\eta \rightarrow {\overline{m}}$ in $C^0({\overline{\omega }}; {\mathcal {Y}})$, as $\eta \rightarrow 0^+$. In turn, setting ${\widehat{m}}_\eta :={\overline{m}}_\eta - ({\overline{m}}_\eta \cdot m_0)m_0$, we have ${\widehat{m}}_\eta \in C^1({\mathbb {R}}^2; {\mathcal {Y}})$ and ${\widehat{m}}_\eta (x', \cdot )\cdot m_0(x')\equiv 0\text { for all }x'$. Moreover, using the third equation in (3.30) in $Q\times (0,M)$ one sees that ${\widehat{m}}_\eta \rightarrow {\overline{m}} - ({\overline{m}} \cdot m_0)m_0={\overline{m}}$ in $C^0({\overline{\omega }};{\mathcal {Y}})$ as $\eta \rightarrow 0^+$. Owing to the latter convergence property and recalling (3.31), we easily deduce

$$\begin{aligned}&\int _{\omega }g_{\mathrm {hom}}(\nabla _{x'} m_0)\, \mathrm{d}x'=\int _\omega \int _{Q_{f_1,f_2}} \Bigl [ | \nabla _{x'} m_{0}(x')+\nabla _{y'}{\overline{m}} (x',y) |^2+ |\partial _{y_3}{\overline{m}} (x', y) |^2\Bigr ]\, \mathrm{d}y \mathrm{d}x'\\&= \lim _{\eta \rightarrow 0^+} \int _\omega \int _{Q_{f_1,f_2}} \Bigl [ | \nabla _{x'} m_{0}(x')+\nabla _{y'}{\widehat{m}}_\eta (x',y) |^2+ |\partial _{y_3}{\widehat{m}}_\eta (x', y) |^2\Bigr ]\, \mathrm{d}y \mathrm{d}x'\\&\ge \inf \biggl \{ \int _\omega \int _{Q_{f_1,f_2}} \Bigl [ | \nabla _{x'} m_{0}(x')+\nabla _{y'}m(x',y) |^2+ |\partial _{y_3}m(x', y) |^2\Bigr ]\, \mathrm{d}y \mathrm{d}x': \\&\quad m\in C^1({\overline{\omega }}; {\mathcal {Y}} )\text { s.t. } m(x', \cdot )\cdot m_0(x')\equiv 0\text { for all }x'\in \omega \biggr \}\,. \end{aligned}$$

Since the other inequality is trivial, this concludes the proof of the lemma. $\square$

We are now ready to establish the upper bound for the limiting exchange energy.

#### Proposition 3.21

Let $m_0\in H^1(\omega ; {\mathbb {S}}^2)$. Then, there exists $\{m_\varepsilon \}_{\varepsilon >0}$ such that $m_\varepsilon \in H^1(\Omega _\varepsilon ; {\mathbb {S}}^2)$ for every $\varepsilon >0$, (3.25) holds and

$$\begin{aligned} \limsup _{\varepsilon \rightarrow 0} \int _{\Omega _\varepsilon } \left( | \nabla _{x'} m_\varepsilon |^2 + \frac{1}{\varepsilon ^2} |\partial _{x_3} m_\varepsilon |^2 \right) \, \mathrm{d}x\le \int _{\omega }g_{\mathrm {hom}}(\nabla _{x'} m_0)\, \mathrm{d}x'\,. \end{aligned}$$

#### Proof

We start by assuming that $m_0\in C^1({\overline{\omega }}; {\mathbb {S}}^2)$. Fix $\eta >0$. Then, by Lemma 3.20 we may find $m\in C^1({\overline{\omega }}; {\mathcal {Y}})$ such that

3.32 $$\begin{aligned} m(x', \cdot )\cdot m_0(x')=0\text { in} Q\times (0,M)\qquad \text {for all }x'\in \omega \end{aligned}$$

and

3.33 $$\begin{aligned}&\int _\omega \int _{Q_{f_1,f_2}} \Bigl [ | \nabla _{x'} m_{0}(x')+\nabla _{y'}m(x',y) |^2+ |\partial _{y_3}m(x', y) |^2\Bigr ]\, \mathrm{d}y \mathrm{d}x'\nonumber \\&\quad \le \int _{\omega }g_{\mathrm {hom}}(\nabla _{x'} m_0)\, \mathrm{d}x'+\eta \,. \end{aligned}$$

For every $\varepsilon >0$ and for $x\in \Omega ^M:=\omega \times (0,M)$, we set

3.34 $$\begin{aligned} {\widehat{m}}_\varepsilon (x):=m_0(x')+\varepsilon m\Bigl (x', \frac{x'}{\varepsilon }, x_3\Bigr ) \qquad \text {and}\qquad m_\varepsilon :=\frac{{\widehat{m}}_\varepsilon }{|{\widehat{m}}_\varepsilon |} . \end{aligned}$$

Clearly $\{m_\varepsilon \}\subset H^1(\Omega ^M; {\mathbb {S}}^2)$ and satisfies (3.25). Since by (3.32) we have $|{\widehat{m}}_\varepsilon |\ge 1$, it is immediately checked that

$$\begin{aligned} | \nabla _{x'} m_\varepsilon |^2 + \frac{1}{\varepsilon ^2} |\partial _{x_3} m_\varepsilon |^2\le | \nabla _{x'}{\widehat{m}}_\varepsilon |^2 + \frac{1}{\varepsilon ^2} |\partial _{x_3} {\widehat{m}}_\varepsilon |^2\,. \end{aligned}$$

Thus, setting $g(x_3, y'):=\chi _{(f_1(y'), f_2(y'))}(x_3)$, we may estimate

$$\begin{aligned} \limsup _{\varepsilon \rightarrow 0}&\int _{\Omega _\varepsilon } \left( | \nabla _{x'} m_\varepsilon |^2 + \frac{1}{\varepsilon ^2} |\partial _{x_3} m_\varepsilon |^2 \right) \, \mathrm{d}x \\&\le \lim _{\varepsilon \rightarrow 0}\int _{\Omega ^M}g\Bigl (x_3, \frac{x'}{\varepsilon }\Bigr )\left( | \nabla _{x'}{\widehat{m}}_\varepsilon |^2 + \frac{1}{\varepsilon ^2} |\partial _{x_3} {\widehat{m}}_\varepsilon |^2\right) \, \mathrm{d}x\\&= \lim _{\varepsilon \rightarrow 0}\int _{\Omega ^M}g\Bigl (x_3, \frac{x'}{\varepsilon }\Bigr )\left( \Bigl | \nabla _{x'}m_0(x')+ \nabla _{y'}m\Bigl (x', \frac{x'}{\varepsilon }, x_3\Bigr )\Bigr |^2 + \Bigr |\partial _{y_3} m\Bigl (x', \frac{x'}{\varepsilon }, x_3\Bigr )\Bigr |^2\right) \, \mathrm{d}x\\&= \int _{\Omega ^M}\int _{Q}g(x_3, y)\left( | \nabla _{x'}m_0(x')+ \nabla _{y'}m(x', y', x_3)|^2 + |\partial _{y_3} m(x', y', x_3)|^2\right) \,\mathrm{d}y' \mathrm{d}x\\&\le \int _{\omega }g_{\mathrm {hom}}(\nabla _{x'} m_0)\, \mathrm{d}x'+\eta \end{aligned}$$

where the last equality has been obtained by passing to the two-scale limit, while the last inequality is (3.33). By the arbitrariness of $\eta$ and a standard diagonalization argument the thesis of the proposition is established when $m_0\in C^1({\overline{\omega }}; {\mathbb {S}}^2)$.

Let now $m_0\in H^1( \omega ; {\mathbb {S}}^2)$. Then there exists $\{m_k\}\subset C^1({\overline{\omega }}; {\mathbb {S}}^2)$ such that $m_k\rightarrow m_0$ in $H^1( \omega ; {\mathbb {S}}^2)$ as $k\rightarrow \infty$. In particular, recalling that $g_{\mathrm {hom}}$ is continuous and $g_{\mathrm {hom}}(\xi )\le |\xi |^2$ (see Remark 3.19), we have

$$\begin{aligned} \int _{\omega }g_{\mathrm {hom}}(\nabla _{x'} m_k)\, \mathrm{d}x'\rightarrow \int _{\omega }g_{\mathrm {hom}}(\nabla _{x'} m_0)\, \mathrm{d}x'\,. \end{aligned}$$

The thesis follows by applying the first part of the proof to each $m_k$ and using diagonalization argument. $\square$

### $\Gamma$-Convergence

In this section, we prove the main compactness and $\Gamma$-convergence theorem by combining all the previous results.

#### Proof of Theorem 2.2

We start by showing part (i). Let $\{m_\varepsilon \}$ be as in the statement and for every $\varepsilon >0$ let ${\overline{M}}_\varepsilon$ be the function in $H^1(\omega ; {\mathbb {R}}^3)$, with $|{\overline{M}}_\varepsilon |\le 1$ defined by

$$\begin{aligned} {\overline{M}}_\varepsilon (x'):=-\int _{f_1(x'/\varepsilon )}^{f_2(x'/\varepsilon ) }m_\varepsilon (x', x_3)\, \mathrm{d}x_3=-\int _{\varepsilon f_1(x/\varepsilon ')}^{\varepsilon f_2(x'/\varepsilon ) }M_\varepsilon (x', x_3)\, \mathrm{d}x_3\,, \end{aligned}$$

where, we recall, $M_\varepsilon (x', x_3):=m_\varepsilon (x', x_3/\varepsilon )$. Note that, in particular, (3.22) holds. Using (3.22), it is straightforward to check that $\{{\overline{M}}_\varepsilon \}$ is bounded in $H^1(\omega ; {\mathbb {R}}^3)$. Thus, up to a (not relabeled) subsequence there exists $m_0\in H^1(\omega ; {\mathbb {R}}^3)$ such that ${\overline{M}}_\varepsilon \rightharpoonup m_0$ weakly in $H^1(\omega ; {\mathbb {R}}^3)$. Observe now that by the one-dimensional Poincaré-Wirtinger’s inequality we have

$$\begin{aligned} \int _{\Omega _\varepsilon }|m_\varepsilon -{\overline{M}}_\varepsilon |^2\, \mathrm{d}x=\int _{\omega }\int _{f_1(x'/\varepsilon )}^{f_2(x'/\varepsilon )}\biggl |m_\varepsilon (x', x_3)--\int _{f_1(x'/\varepsilon )}^{f_2(x'/\varepsilon )}m_\varepsilon (x', x_3)\, \mathrm{d}x_3\biggr |^2\, \mathrm{d}x'\\ \le \int _\omega \frac{(f_2(x'/\varepsilon )-f_1(x'/\varepsilon ))^2}{\pi ^2} \int _{f_1(x'/\varepsilon )}^{f_2(x'/\varepsilon )}|\partial _{x_3} m_\varepsilon |^2\, \mathrm{d}x_3\mathrm{d}x'\le C\varepsilon ^2\,, \end{aligned}$$

thanks to (3.22), for some constant $C>0$ independent of $\varepsilon$. We deduce that

$$\begin{aligned} \int _{\Omega _\varepsilon }|m_\varepsilon -m_0|^2\, \mathrm{d}x\rightarrow 0\,. \end{aligned}$$

For part (ii), we may assume without loss of generality that

$$\begin{aligned} \liminf _\varepsilon E_\varepsilon (m_\varepsilon )=\lim _\varepsilon E_\varepsilon (m_\varepsilon )<+\infty \,, \end{aligned}$$

In particular, (3.22) holds. Thus, defining ${\overline{M}}_\varepsilon$ as before, we deduce that $\{{\overline{M}}_\varepsilon \}$ is bounded in $H^1$. By (2.10) it readily follows that

3.35 $$\begin{aligned} {\overline{M}}_\varepsilon \rightharpoonup m_0\qquad \text {weakly in }H^1(\omega ; {\mathbb {R}}^3). \end{aligned}$$

In turn, by Lemma 3.2 (and Remark 3.3) and Proposition 3.14 we get

3.36 $$\begin{aligned} \frac{1}{\varepsilon }\int _{{\mathbb {R}}^3}|\nabla u_\varepsilon |^2\, \mathrm{d}x\rightarrow \int _\omega A_{\mathrm {hom}}\, m_0\cdot m_0\, \mathrm{d}x'\,, \end{aligned}$$

which together with Proposition 3.16 yields the conclusion of part (ii).

Part (iii) easily follows from Proposition 3.21 and the fact that (3.36) holds whenever (2.10) and (3.22) hold. $\square$

#### Proof of Corollary 2.3

By Theorem 2.2 and standard $\Gamma$-convergence arguments, we infer that there exists a global minimizer $m_0$ of $E_0$ in $H^1(\omega ; {\mathbb {S}}^2)$ such that, up to a (not relabeled) subsequence, (2.10) holds. It is now easy to see that $m_0$ is a global minimizer if and only if it is constant and minimizes the quadratic form associated to the matrix $A_{\mathrm {hom}}$. This concludes the proof of the corollary. $\square$

#### Remark 3.22

The result of Corollary 2.3, together with the proof of the upper bound (see (3.34)), suggests the following two-scale expansion for the minimizers $m_\varepsilon$:

$$\begin{aligned} m_\varepsilon (x)\approx e_0+\varepsilon m\Bigl (x', \frac{x'}{\varepsilon }, x_3\Bigr ) \end{aligned}$$

for suitable function *m*, *Q*-periodic in the second variable.
